# Three‐Step Pulse Strategy Enhances Ultradilute Nitrate‐to‐Ammonia Conversion via Microenvironment and Mass Transfer Control

**DOI:** 10.1002/advs.202507720

**Published:** 2025-07-28

**Authors:** Kouer Zhang, Gang Liu, Qing Wang, Xiaoyu Huo, Xiaohong Zou, Mingcong Tang, Xiao Zhang, Liang An

**Affiliations:** ^1^ Department of Mechanical Engineering The Hong Kong Polytechnic University Hung Hom Kowloon Hong Kong SAR 999077 China; ^2^ Research Institute for Smart Energy The Hong Kong Polytechnic University Hung Hom Kowloon Hong Kong SAR 999077 China

**Keywords:** electrocatalysis, nitrate reduction, wastewater treatment

## Abstract

Nitrate pollution in wastewater, primarily originating from industrial, agricultural, and domestic sources, typically occurs at concentrations of 10 mm or lower. Although the nitrate reduction reaction (NO_3_RR) has been one of the most intensively researched fields with a mature FE (Faraday efficiency) over 90% and a milligram‐level yield of ammonia, it remains difficult to deal with low‐nitrate environments, such as municipal wastewater. In this study, a three‐step pulsed strategy is presented that attains nearly 100% ammonia FE from an ultralow 10 mm nitrate concentration electrolyte, representing a threefold enhancement over the conventional constant potentiostatic approach. Through operando characterizations, density functional theory calculations and COMSOL simulations, the mechanism is elucidated by which various potential biases concurrently modulate NO_3_RR intermediates, thereby enhancing reaction kinetics and effectively suppressing the competing hydrogen evolution reaction. Furthermore, practical application in a flow cell, along with techno‐economic analysis, highlights the technological and economic feasibility of converting nitrate into valuable ammonia directly from wastewater without preconcentration. The research advances the understanding of pulse‐driven strategies and the modulation of ionic microenvironments in electrochemical processes, paving the way for practical and environmentally friendly wastewater treatment and ammonia synthesis.

## Graphical abstract

Electrochemical treatment of low‐concentration nitrate in wastewater is challenging. This study introduces a three‐step pulsed method that achieves near‐perfect efficiency in converting ultradilute nitrate to ammonia. Combined experiments and simulations reveal how pulsed strategy boost kinetics while suppressing competing reactions. Continuous production and economic analysis confirm the feasibility of direct nitrate‐to‐ammonia conversion from wastewater, advancing sustainable water treatment technology.

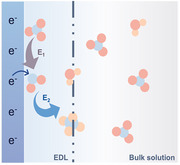

## 1 Introduction

Excessive nitrate ions (NO_3_^−^), primarily from the overuse of nitrogen‐based fertilizers and sewage discharge, have become one of the most common water pollutants worldwide. This pollution could significantly disrupt the natural nitrogen cycle.^[^ ^1^, ^2^ ^]^ Electrochemical reduction of nitrate ions to ammonia (NH_3_), powered by renewable energy, has emerged as a promising process for waste treatment and recovery from wastewater with energy and environmental sustainability.^[^ ^3^ ^]^ Particularly, this method presents a more sustainable alternative to the traditional Haber–Bosch process, which is energy‐intensive (up to 2% of anthropogenic energy) and contributes ≈1% of global carbon dioxide (CO_2_) emissions.^[^ ^4^, ^5^ ^]^ In recent years, considerable efforts and progress have been made in the nitrate reduction reaction (NO_3_RR) toward ammonia production, particularly in the catalyst design. Numerous works have demonstrated high Faraday efficiencies (FE; e.g., over 90%) and mg‐level production rate at commercial current densities.^[^ ^6^, ^7^, ^8^, ^9^ ^]^ However, these performance metrics have largely been achieved using high‐concentration nitrate electrolytes (>100 mm). While these results are promising, they do not fully address the challenges posed by nitrate contamination in real‐world scenarios, where nitrate often exists at much lower concentrations. For example, in agricultural runoff and municipal wastewater, nitrate levels often range from 10 to 50 mg‐N L^−1^.^[^ ^10^ ^]^ Therefore, developing technologies capable of efficient conversion of low‐concentration nitrate to ammonia is essential. In our former work, we developed an interfacial electric field to modulate the local nitrate concentration, thus solving the low‐concentration dilemma.^[^ ^11^ ^]^

Moreover, it has been widely reported that the NO_3_RR involves a tandem process of nitrate‐to‐nitrite (NO_3_^−^‐to‐ NO_2_^−^) step and nitrite‐to‐ammonia (NO_2_^−^‐to‐ NH_3_) step.^[^ ^12^, ^13^, ^14^ ^]^ Generally, the NO_3_^−^‐to‐ NO_2_^−^ step is regarded as the rate‐determining step (RDS) of NO_3_RR due to the sluggish kinetics, leading to insufficient NO_2_^−^ formation as the key intermediate. Although novel catalysts could enhance the reaction kinetics, it is hard to regulate the scaling relationship between the tandem steps. Besides, considering the negative potential applied during the NO_3_RR process, NO_3_^–^ ions are repelled from the cathode, further reducing their concentration at the catalyst surface. Under the condition of ultralow nitrate concentration, these dilemmas would be deepened along with more intense hydrogen evolution reaction (HER) as the side reaction, leading to poor ammonia Faraday efficiency and limited yield rate.^[^ ^15^ ^]^ To address the challenges of low activity and selectivity in converting low concentrations of NO_3_^−^ to NH_3_, a promising approach involves modulating the ionic microenvironment at the electrode–electrolyte interface.^[^ ^16^ ^]^ This technique offers intrinsic advantages by periodically altering the potential or current, which are not accessible under conventional potentiostatic or galvanostatic conditions. Pulse electrolysis has been widely applied in various fields, including hydrogen peroxide production, organic electrosynthesis, and carbon dioxide reduction.^[^ ^17^, ^18^, ^19^ ^]^ Two key functions of this strategy have been identified in previous works: First, it aids in controlling the migration and concentration of charged species within the electrical double layer. By pulsing the reaction during the anodic or resting phase, reactants have the opportunity to diffuse back to the electrode surface, thereby preventing the depletion of reactants near the electrode and reducing mass transport limitations, which are particularly critical in low‐concentration systems. Second, modulating the pulse potential allows for the fine‐tuning of reaction selectivity.^[^ ^20^ ^]^ For instance, pulses at specific potentials can promote the formation of surface species that favor the specific reaction pathways, regulating the crucial intermediates and suppressing the side reactions.

Cu‐based catalysts have emerged as a promising class for NO_3_RR due to the comparable energy levels between the d‐orbitals of copper and the lowest unoccupied molecular orbital (LUMO π*) of nitrate, coupled with their relatively low HER activity.^[^ ^21^, ^22^, ^23^ ^]^ Nonetheless, Cu usually encounters a substantial energy barrier during the hydrogenation process of the NO_3_^−^‐to‐ NO_2_^−^ step, limiting the generation of *NO_2_^−^ intermediate. To overcome this challenge, we present a novel strategy to directly modulate the ionic microenvironment at the electrode–electrolyte interface. Our strategy is to achieve high selectivity and activity using a three‐step pulse reaction pathway: i) accumulation of adsorbed NO_3_^−^ at the cathode surface using positive potential pulses; ii) subsequent optimization of *NO_2_^−^ production by selectively converting NO_3_^−^ to NO_2_^−^ using mild negative pulses at low kinetic barriers; and iii) rapid hydrogenation of accumulated *NO_2_^−^ to NH_3_ using strong negative pulses. This approach effectively separates the kinetic mismatch steps, bypasses the rate‐limiting NO_3_^−^ activation barrier, and achieves temporal and spatial control over the concentration of *NO_2_^−^ intermediates. As a result, it maximizes selectivity toward NH_3_ and enhances mass transfer even in ultradilute nitrate solutions (≤10 mm). Therefore, this opens a significant pathway to obtaining nitrate with ultralow cost, while also leading to necessary intermediates at the optimal generated ratio, thus enhancing the overall conversion efficiency. In the three‐step pulsed process, the first step involves applying a positive potential at the cathode to accumulate adsorbed NO_3_^−^ on the electrode surface. The second step applies a small negative bias to drive the conversion of NO_3_^−^ to NO_2_^−^, producing enough of the NO_2_^−^ intermediate. In the third step, a large negative potential is applied to promote the rapid conversion of accumulated NO_2_^−^ to NH_3_. Here, we tested and verified this approach under ultralow nitrate concentration (≤10 mm) by applying a Cu nanowire/Cu foam (Cu NW/Cu foam) electrode. Our experimental results indicate that the three‐step pulsed strategy significantly improves NH_3_ selectivity. Specifically, ammonia Faraday efficiency increased to 99.84% at −0.7 V versus reversible hydrogen electrode (RHE), presenting a threefold enhancement compared to the constant potentiostatic methods. Furthermore, theoretical simulations strongly indicate that NO_3_^−^ is enriched at positive bias potentials, and the localized accumulation‐depletion process of the intermediate NO_2_^−^ during the multipulsed steps is also revealed.

## 2 Results and Discussion

### 2.1 Synthesis and Characterizations of Cu NW/Cu Foam

**Figure 1:**
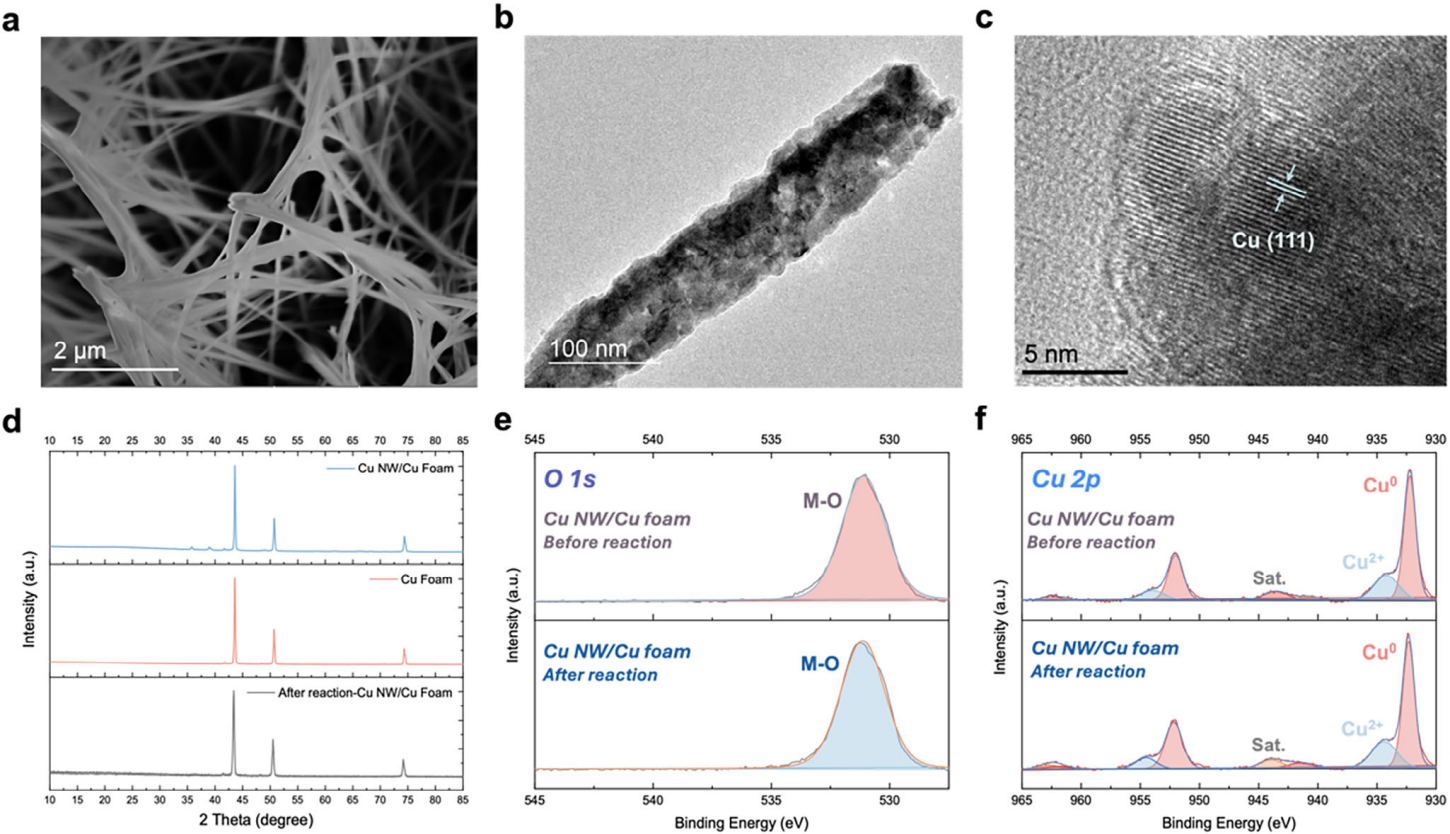
Morphology and structural characterization of Cu NW/Cu foam. a) SEM image. b) TEM image and c) HRTEM image of Cu NW/Cu foam. d) XRD patterns of Cu foam and Cu NW/Cu foam before and after reaction. High‐resolution XPS spectra of e) O1s and f) Cu 2p.

Referring to our previous works, we synthesized the Cu NW/Cu foam through a two‐step green approach, including in situ growth and hydrogen reduction.^[^ ^8^ ^]^ As demonstrated in the scanning electron microscopy (SEM) images, a uniform distribution of Cu NW array with a length of over 2.0 µm could be observed (**Figure** **1**a; and Figures S1 and S2, Supporting Information). The uniform elemental distribution was proved through SEM mappings (Figures S3 and S4, Supporting Information). With the Cu NW array in situ grown on the Cu foam substrate, the hydrophilicity was also prominently improved and could be proved through the contact angle test (Figure S5, Supporting Information). The untreated Cu foam showed hydrophobic characteristics with an average contact angle of 136.2°, while the Cu NW/Cu foam electrode became super hydrophilic with a contact angle of 0°. The high surface area, porous network of Cu NWs creates capillary forces and enhances effective surface area, promoting water penetration via Wenzel‐state wetting.^[^ ^11^, ^24^ ^]^ The structure of the Cu NW was better visualized through transmission electron microscopy (TEM), indicating a diameter of 100 nm (Figure 1b). High‐resolution transmission electron microscopy (HRTEM) provided the direct image of the lattice structure of the Cu NW sample, of which Cu (111) occupied the main status with a lattice spacing of 0.208 nm (Figure 1c). X‐ray diffraction (XRD) and X‐ray photoelectron spectroscopy (XPS) were carried out to further unveil the chemical composition of Cu NW (Figure 1d–f). The electrochemical surface area (ECSA) of Cu NW/Cu foam was detected based on the circuit voltammetry (CV) method (Figure S7, Supporting Information). Due to the inevitable surface oxidation of samples in the air, the weak CuO oxidation peaks appearing at 35.5°, 38.9°, and 42.2° could be observed in XRD patterns. We examined the sample after a long‐term stability test for 120 000 s (the experimental details are discussed later in pulsed performance evaluation), and the surface oxidized state was largely diminished as shown in Figure 1d. As depicted in the high‐resolution XPS spectra of Cu 2p (Figure 1f), the peaks for CuO are located at 934.2 eV (Cu^2+^ 2p_3/2_) and 954.2 eV (Cu^2+^ 2p_1/2_), along with the shake‐up satellite peaks in both samples. The presence of Cu^2+^ was caused by the surface oxidation from the air and the real active site for NO_3_RR has been proven to be metallic Cu previously.^[^ ^8^ ^]^ Consequently, Cu^0^ peaks appear at 932.4 eV (Cu^0^ 2p_3/2_) and 952.3 eV (Cu^0^ 2p_1/2_) in Cu NW.^[^ ^25^, ^26^ ^]^ Furthermore, the Cu NW/Cu foam electrode after the reaction was examined. A slight composition shift of Cu^2+^ to Cu° could be observed according to the XPS composition analysis of Cu 2p spectra, in correspondence with the diminishment of CuO peaks in XRD (Tables S1 and S2, Supporting Information). Moreover, based on the XPS survey and Cu LMM spectra, the composition of Cu NW remains consistent after the reaction, indicating its good stability in the NO_3_RR process (Figure S6, Supporting Information).

### 2.2 NO_3_RR and NO_2_RR Performance Evaluation of Cu NW/Cu Foam

**Figure 2:**
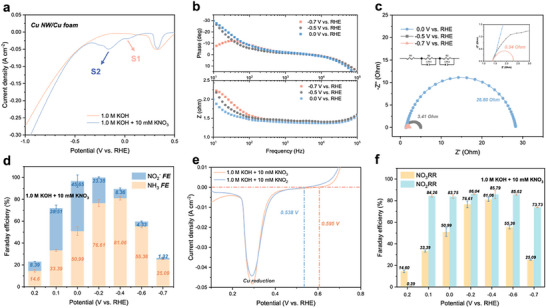
Electrocatalytic performance evaluation of Cu NW/Cu foam. a) LSV curves of NO_3_RR and HER. b) Bode plots and c) Nyquist plots of NO_3_RR under different potentials (−0.7/−0.5/0.0 V vs RHE). d) Faraday efficiency of NO_2_^−^ and NH_3_ under different potentials of NO_3_RR. e) The onset points for NO_3_RR and NO_2_RR of LSV curves. f) Ammonia Faraday efficiency of NO_3_RR and NO_2_RR in 10 mm KNO_3_ and KNO_2_, respectively.

Initially, the electrochemical performance was conducted in an H‐cell with an anion exchange membrane. The samples fabricated here will be used as the working electrode (cathode) in this three‐electrode system, while a Pt foil and an Hg/HgO electrode in 1.0 m KOH solution will act as the counter electrode (anode) and reference electrode, respectively. The catholyte will be made up of 1.0 m KOH with KNO_3_ varying from 1.0 to 10.0 mm, while the anolyte will be 1.0 m KOH unless stated otherwise. Electrochemical performance data were collected and analyzed using an electrochemical workstation. The corresponding FEs were calculated based on the concentration detected from the electrolyte as depicted in the notes and figures of the Supporting Information (Figure S11, Supporting Information). To thoroughly investigate the effects of the three‐step pulsed method on NO_3_^−^ to NH_3_ conversion, we systematically studied the applied voltage and duration by steps. First, we explored the conventional NO_3_RR under potentiostatic conditions. The linear sweep voltammetry (LSV) curves could reveal the intrinsic activity of NO_3_RR. It was presented that the Cu NW/Cu foam electrode exhibited a current density gap when comparing the electrolyte with or without 10 mm KNO_3_, indicating the presence of activated NO_3_RR (**Figure** **2**a). Especially, the downward peaks appearing at 0.1 V versus RHE and −0.2 V versus RHE represented the two stages of the NO_3_RR process as listed below^[^ ^27^, ^28^ ^]^

(1) $$S1:N{O_{3}}^{-}+H_{2}O+2e^{-}\to N{O_{2}}^{-}+2OH^{-}$$

(2) $$S2:N{O_{2}}^{-}+5H_{2}O+6e^{-}\to NH_{3}+7OH^{-}$$

Generally, it has been widely reported that the S1 is the RDS of NO_3_RR, meaning that the kinetics of NO_3_^−^ to NO_2_^−^ step would significantly impact the overall conversion rate to ammonia. Furthermore, LSV analysis of commercial Cu foam in 1.0 m KOH with 10 mm KNO_3_ (Figure S9, Supporting Information) reveals fundamental catalytic differences. While characteristic nitrate reduction peaks are observable, their significant negative shift compared to Cu NW/Cu foam indicates slower NO_3_RR kinetics due to reduced active sites and poorer mass transfer. Critically, the sharp reduction peak at around 0.3 V versus RHE corresponds to Cu^2+^/Cu^+^ reduction, exposing irreversible surface oxidation and reconstruction during operation.^[^ ^29^ ^]^ We also performed electrochemical impedance spectroscopy (EIS) measurements to evaluate the reaction kinetics of Cu NW/Cu foam at different applied potentials.^[^ ^30^ ^]^ Impedance data were acquired from the equivalent circuit model (Figure 2c), where *R*_s_, *R*_c_, and *R*_m_ are the resistances of the solution, interfacial charge transfer, and mass transfer, respectively, and with two constant phase elements CPE1 and CPE2 to demonstrate the electrical double layers. According to the Bode plots (Figure 2b), the peak appearing in the frequency range of 10^1^–10^2^ Hz at −0.7 V versus RHE suggested that the rapid HER was the side reaction under this potential.^[^ ^31^ ^]^ To quantitatively probe interfacial processes, we conducted electrochemical impedance spectroscopy (EIS) across key potentials. The Nyquist plots (Figure 2c) were modeled using an equivalent circuit accounting for solution resistance (*R*_s_), charge transfer resistance (*R*_ct_), and mass transfer resistance (*R*_mt_). At the optimal ammonia production potential (−0.7 V vs RHE), charge transfer resistance (*R*_ct_) drops dramatically to 0.54 Ω, which is 50‐fold lower than at 0.0 V, confirming drastically accelerated charge transfer during NO_3_RR. To clearly distinguish the directions of the NO_3_RR reaction and the side HER, we investigated the product selectivity through the quantitative analysis of NH_3_ and NO_2_^−^ under a series of bias potentials from 0.2 to −0.7 V versus RHE (Figure 2d). Specifically, the FE for NO_2_^−^ reached a peak of 45.65% at 0.0 V versus RHE and gradually decreased toward the negative potential. Meanwhile, with the decline in NO_2_^−^ FE, the NH_3_ FE gradually increased until −0.4 V versus RHE. Under more negative potentials, the production of NH_3_ and NO_2_^−^ was suppressed. At −0.7 V versus RHE, the FE of NH_3_ and NO_2_^−^ were only 25.09% and 1.32%, respectively. This phenomenon indicated the intensification of the side HER with the negative shift of the bias potential. To be mentioned, the low FE for NH_3_ and NO_2_^−^ at 0.2 V were caused by the reduction of CuO at the electrode surface which could also be verified through the high overlap of LSV curves in either electrolyte with or without nitrate. Herein, we could speculate that under a relatively positive potential range (0.0–0.1 V), the NO_3_^−^ to NO_2_^−^ step functioned as the prominent reaction, while the NO_2_^−^ to NH_3_ step was not as active as expected. Under reductive bias potentials, the NO_2_^−^ to NH_3_ step was accelerated, boosting the ammonia yield rate and efficiency but still facing the threat from the competitive HER. To create an intuitive simulation of the two steps in the NO_3_RR process, an electrolyte containing 10 mm KNO_2_ was used for comparison (Figure S10, Supporting Information). The zoomed‐in LSV curves revealed the onset points for NO_3_RR and NO_2_RR at 0.595 and 0.538 V versus RHE, respectively, highlighting the initial reduction stages of NO_3_^−^ and NO_2_^−,^ while the redox peak for Cu could be observed at around 0.3 V versus RHE (Figure 2e). The difference between these onset points indicated the distinct potentials required for the conversion of NO_3_^−^ to NO_2_^−^ and NO_2_^−^ to NH_3_. Moreover, we investigated the NH_3_ FE for NO_2_RR to simulate the subsequent step of converting NO_2_^−^ to NH_3_. Notably, it was observed that once the RDS was surpassed, the FE for the conversion of NO_2_^−^ to NH_3_ remained stable up to −0.7 V versus RHE, indicating the great conversion activity of Cu from ammonia. Beyond this potential, the competing HER would start to affect the NO_2_RR process. Herein, we have determined that the RDS (NO_3_^−^ to NO_2_^−^) significantly limited the efficiency of ammonia production. This finding is crucial for addressing the challenges associated with NO_3_RR at ultralow nitrate concentrations. Overcoming this bottleneck is essential for enhancing the overall performance of the ammonia production process.

### 2.3 Pulsed NO_3_RR Performance Evaluation

**Figure 3:**
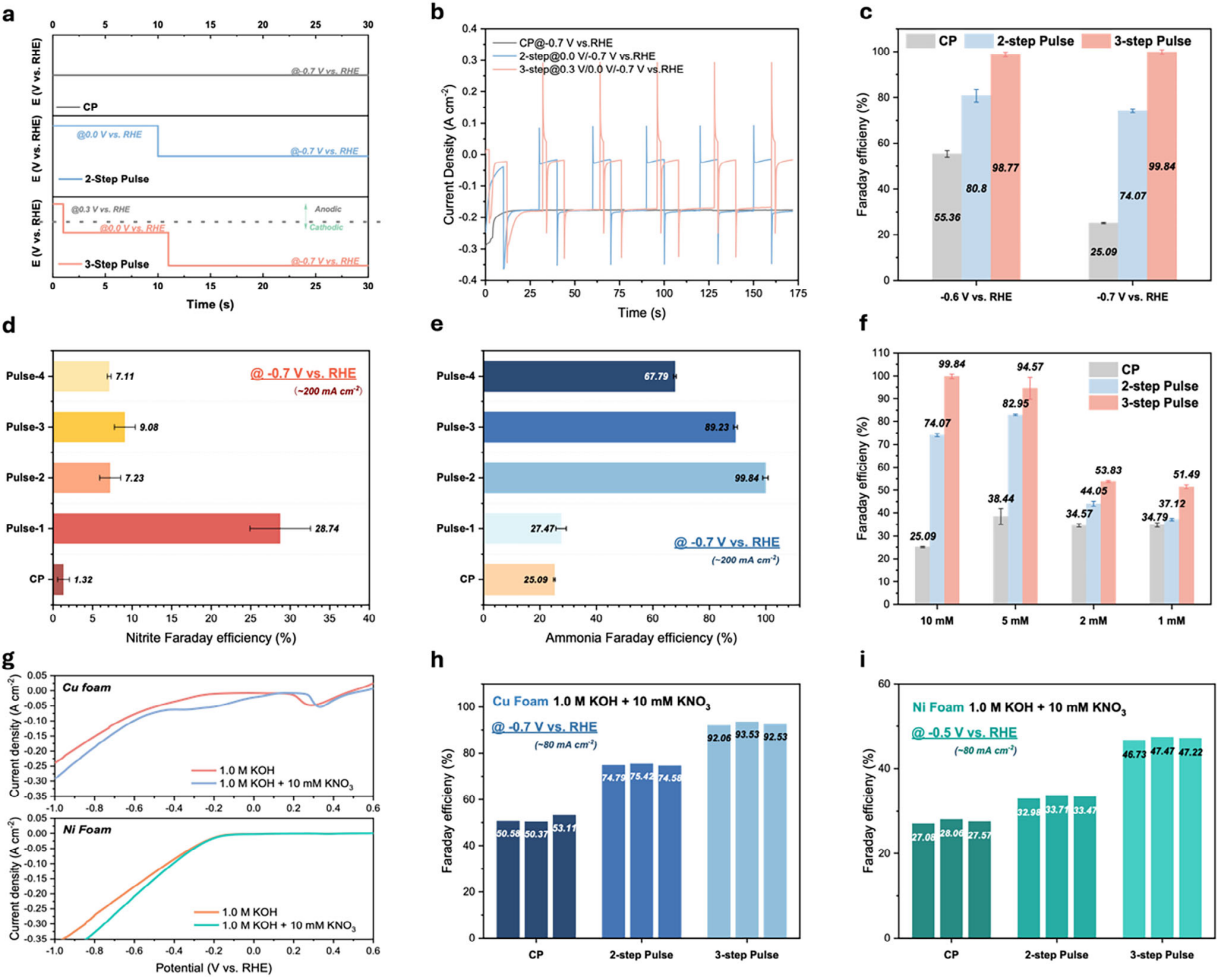
NO_3_RR performance of 3‐step pulse electroreduction. a) Schematic of *V*–*t* relationships of CP, 2‐step pulse and 3‐step pulse NO_3_RR. b) *I*–*t* curves of the corresponding pulsed NO_3_RR process. c) Ammonia Faraday efficiency of CP, 2‐step pulse and 3‐step pulse NO_3_RR at −0.6  and −0.7 V versus RHE. d) Nitrite Faraday efficiency and e) ammonia Faraday efficiency at −0.7 V versus RHE under different pulsed conditions. f) Ammonia Faraday efficiency of the 3‐step pulse NO_3_RR in different concentrations of KNO_3_. g) LSV curves of HER and NO_3_RR on Cu foam and Ni foam. Ammonia Faraday efficiency of CP, 2‐step pulse and 3‐step pulse NO_3_RR of commercial h) Cu foam and i) Ni foam.

Aiming at solving the RDS obstacle, especially under ultralow nitrate concentration, two principal concerns should be addressed: 1) the inadequate localized nitrate concentration at the catalyst surface, and 2) the sluggish kinetics of the nitrate‐to‐nitrite conversion process. In response to these challenges, we propose an innovative three‐step pulsed method designed to modulate the ionic microenvironment at the electrode–electrolyte interface. This method not only facilitates the accumulation of nitrate but also promotes the formation of essential intermediates at an optimal generation ratio, enhancing the overall efficiency of the conversion process. In the three‐step pulsed process, the first step involves applying a positive potential (0.3 V vs RHE) at the cathode to accumulate adsorbed NO_3_^−^ on the electrode surface. Here, we set the positive potential time at 1 s due to the dynamic character of Cu surface, which would be easily oxidized under static positive potentials (Figure S8, Supporting Information). The second step applies a small negative bias (0.0 V vs RHE) to drive the conversion of NO_3_^−^ to NO_2_^−^, producing enough of the NO_2_^−^ intermediate. The selection of the potential for the second step was informed by previous tests evaluating the FE of nitrite. In the third step, negative potentials were applied to promote the rapid conversion of accumulated NO_2_^−^ to NH_3_ (**Figure** **3**a). As a comparison, we investigated the 2‐step pulsed strategy, which only included the second and third steps. The corresponding *I*−*t* curves are presented in Figure 3b. The NH_3_ FE of constant potential (CP), 2‐step pulse and 3‐step pulse NO_3_RR were determined through the chromogenic method and compared at −0.6 and −0.7 V versus RHE as the third‐step reductive potentials. As clearly stated in the figure, the FE of ammonia increased from 55.35% under CP conditions to 80.80% under 2‐step pulsed conditions (Figure 3c). The FE continued increasing to 98.77% with the 3‐step pulse at −0.6 V versus RHE. Moreover, at −0.7 V versus RHE, with the current density around 200 mA cm^−2^, the highest NH_3_ FE went through a threefold increasement from 25.09% to nearly 100% at ultralow nitrate concentration of merely 10 mm. To highlight the importance of optimizing nitrite levels for maximizing utilization and enhancing NH_3_ FE, it is crucial to modulate the scaling relationship between the two steps of the NO_3_RR. In this study, we further examine the reaction time for steps 2 and 3, which predominantly govern the S1 and S2 processes of NO_3_RR, respectively. The positive potential time was consistently set at 1 s. For step 2, we implemented a gradient variation in reaction times of 20, 10, 15, and 5 s, while maintaining a constant reaction time of 20 s for step 3. These different time combinations were designated as Pulse‐1, Pulse‐2, Pulse‐3, and Pulse‐4, providing a basis for comparison with the CP reaction. As shown in Figure 3d, the FE of nitrite after a 1000s 3‐step pulsed reaction showed great variations under different step 2 reaction times. When the corresponding reaction time for the three steps was 1s‐20s‐20s (Pulse‐1), high nitrite concentration could be detected in the electrolyte after the reaction, suggesting the excessive generation of NO_2_^−^ and the inadequate conversion from NO_2_^−^ to NH_3_. Meanwhile, Pulse‐2, Pulse‐3, and Pulse‐4 exhibited similar nitrite FE below 10%. The ammonia FE, as depicted in Figure 3e, offers direct insights into the optimized scaling relationship between S1 and S2 that results in the highest NH_3_ FE. Notably, the Pulse‐2 condition achieved the highest NH_3_ FE of 99.84% at −0.7 V versus RHE. Generally, a low ratio of the duration of step 1 to the total process time typically results in insufficient nitrite production, whereas a high ratio can lead to a reduced rate of nitrite conversion to ammonia. The pulsed strategy was applied on even lower nitrate concentrations of 5, 2, and even 1 mm, showing great potential in ultralow nitrate removal (Figure 3f). Moreover, the pulsed strategy is suitable for various commercial electrodes and here we have examined the corresponding NH_3_ FE on Cu foam and Ni foam in 10 mm KNO_3_ at −0.7 V versus RHE (Figure 3g–i). Specifically, although the pulsed strategy significantly improved the performance of Ni foam (increasing FE from 27% to 47%), its fundamental limitations in activating nitrate reduction prevent the formation of clear peaks in LSV curves. These findings show that while pulse optimization enhances kinetics, intrinsic properties ultimately dictate the electrochemical signatures. In addition, isotope‐labeling measurement was also adopted to prove the source and expel contamination of ammonia produced in this work (Figure S12, Supporting Information). We examined the long‐term stability of the 3‐step pulsed NO_3_RR and after 120 000 s under the condition of Pulse‐2 in the electrolyte containing 10 mm KNO_3_, no significant degradation could be observed (Figure S13, Supporting Information). While these results demonstrate promising stability under pulsed operation, industrial deployment will require validation over thousands of hours. This remains a significant area for future scale‐up studies, and considerable work is still needed before industrialization can be achieved.^[^ ^32^, ^33^ ^]^

Given the compositional complexity of real wastewater, the influence of common impurity anions (Cl^−^, SO_4_^2−^) on ammonia Faraday efficiency was evaluated. To assess these impacts, controlled experiments were conducted using synthetic nitrate‐containing wastewater with 10 mm KCl and 10 mm K_2_SO_4_ (representing typical anion concentrations in municipal/industrial effluents). These tests were performed under two‐step/three‐step pulsed conditions to the baseline system (1 m KOH with 10 mm KNO_3_) and the results were presented in Figure S14 (Supporting Information). Here we could find out that no significant inhibition of ammonia production was observed in the presence of Cl^−^, SO_4_^2−^. The pulsed electrochemical strategy could also effectively mitigate competitive HER reaction and thus greatly enhance the ammonia production efficiency.

### 2.4 Mechanism Understanding of Pulsed NO_3_RR

**Figure 4:**
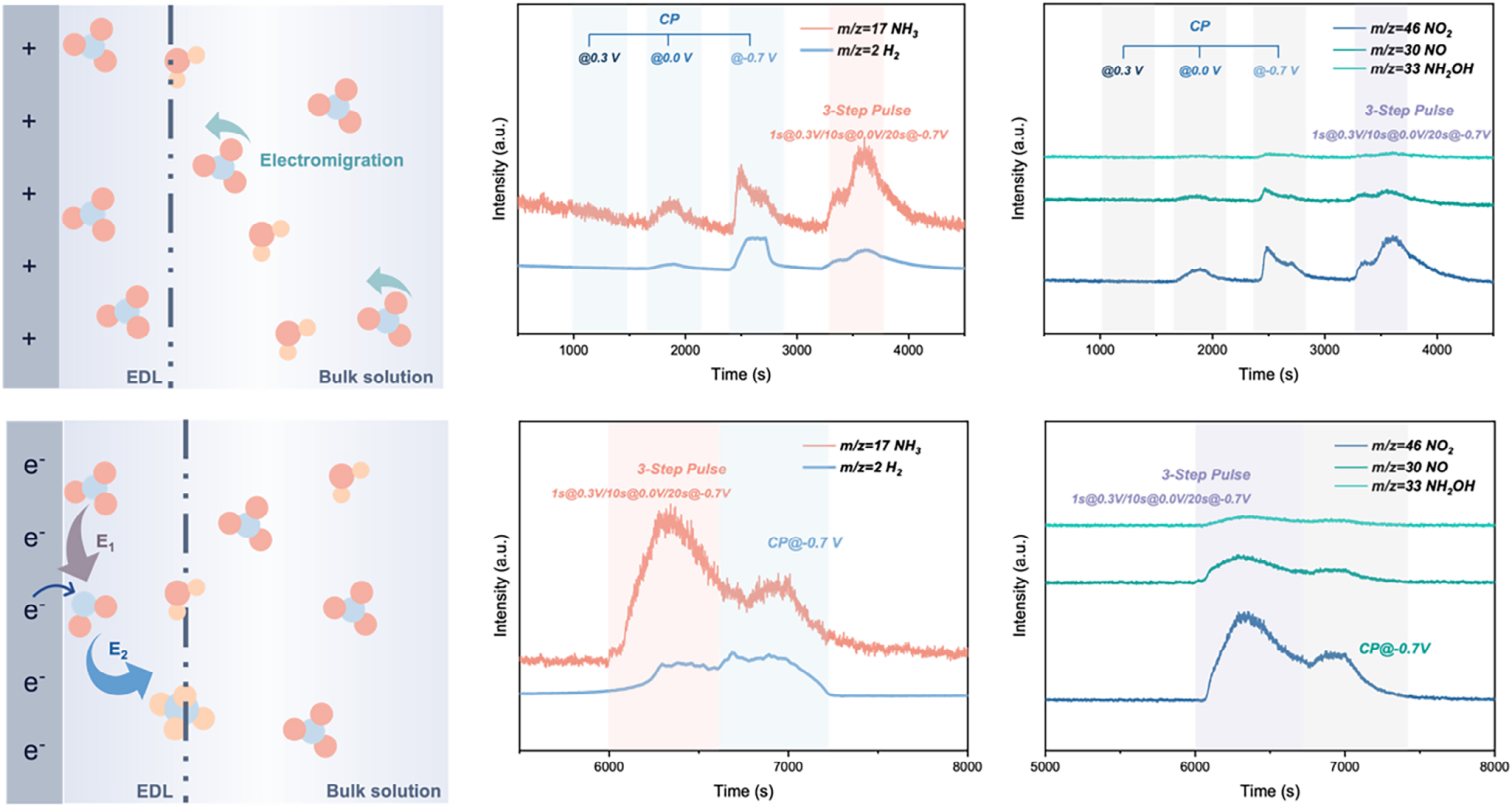
Mechanism analysis of 3‐step pulse electroreduction. Schematic of pulse NO_3_RR at a) anodic potential and b) cathodic potential. DEMS products signals of NH_3_ and H_2_ (m/z = 17 and 2) under c) various potentials (0.3, 0.0, and −0.7 V vs RHE) and d) different reduction conditions (CP and 3‐step pulsed electroreduction). DEMS intermediates signals of NO_2_, NO, and NH_2_OH (m/z = 46, 30, and 33) under e) various potentials and f) different reduction conditions.

Based on the former experimental results, we have proposed the effect of step‐controlled bias potentials on the microenvironment at the electrode surface, influencing the key intermediate NO_2_^−^ generation and the product selectivity. As depicted in **Figure** **4**a, with a positive bias applied to the electrode, the NO_3_^−^ ions are accumulated through the electromigration process from the bulk electrolyte solution. This step solved the problem brought by the ultralow electrolyte concentration as well as possible. Afterward, under the first small reductive potential (E_1_), accumulated nitrate ions were converted into nitrite. The nitrite as the main product is generally formed at a small reductive potential because of the more rapid kinetics of the NO_3_^−^ to NO_2_^−^ step compared to the NO_2_^−^ to NH_2_ step without obvious hydrogen evolution.^[^ ^16^ ^]^ Under the following large reductive potential (E_2_), the intermediate nitrite is continuously converted into ammonia (Figure 4b). In this case, the increased kinetics at E_2_ would lead to the acceleration of the NO_2_^−^ to NH_2_ step. Meanwhile, the kinetics of side HER also increased, while the large amount of nitrite greatly reduces the probability of side effects.

In our work, online differential electrochemical mass spectrometry (DEMS) was employed to monitor the gaseous products and intermediates that evolved during the reaction (Figure 4c–f). DEMS enables real‐time quantitative analysis of volatile species, thereby providing deeper insights into reaction pathways and the efficiency of ammonia production, as well as the capability to analyze additional side reactions. Over time, signals corresponding to m/z values of 2, 17, 28, 30, and 44 are detected, which can be attributed to H_2_, NH_3_, N_2_, NO, and N_2_O, respectively.^[^ ^34^ ^]^ Initially, we investigated the signals of the NO_3_RR products, mainly NH_3_ and H_2_ and a slight amount of N_2_O (Figure S15, Supporting Information). At 0.3 V versus RHE, no signal for NH_3_ and H_2_ could be detected, illustrating the role of this step in a sideways manner. With the negative shift of the potentials to 0.0 and −0.7 V versus RHE, the signal for both NH_3_ and H_2_ became more intensified while H_2_ went through a drastic increment (Figure 4c). After pausing for 1000 s, the 3‐step pulsed strategy was applied and the prominent prohibition of HER could be observed along with the great enhancement in ammonia yield. Then, we compared product signals of the CP condition at −0.7 V and the 3‐step pulsed electroreduction in a steady period of 1000 s. The DEMS result showed an intuitive comparison, suggesting the strong promotion of NO_3_RR toward ammonia along with the inhibition of HER. Besides, some intermediates of NO_3_RR were also examined. To be mentioned, exponential nitrite can be detected with the 3‐step pulse compared to the CP electroreduction, becoming strong evidence for the successful modulation of nitrite as the key intermediate. Furtherly, we applied the in situ Fourier transform infrared spectroscopy (FTIR) to track the absorbed reactants and intermediates at the electrode surface under various potentials. The results suggest the adsorption of the specific ions under the pulse process, which is controlled by potential biases. As shown in the FTIR spectra (Figure S16, Supporting Information), the peaks appearing at around 1180 and 1230 cm^−1^ represent the absorbed NO_2_^−^ and NO_3_^−^, respectively. It could be found out that while the potential bias shifts negatively, the intensity of NO_3_^−^ peaks remains stable from −0.2 to −0.6 V versus RHE, indicating the less attractive to anionic NO_3_^−^. Meanwhile, for the peaks representing NO_2_^−^, the highest intensity appears between −0.2 and −0.4 V versus RHE, which also suggests the strong preference for nitrate to nitrite in our Step 2. Besides, other absorbed species including *NO, *NH_2_OH could be observed.

**Figure 5:**
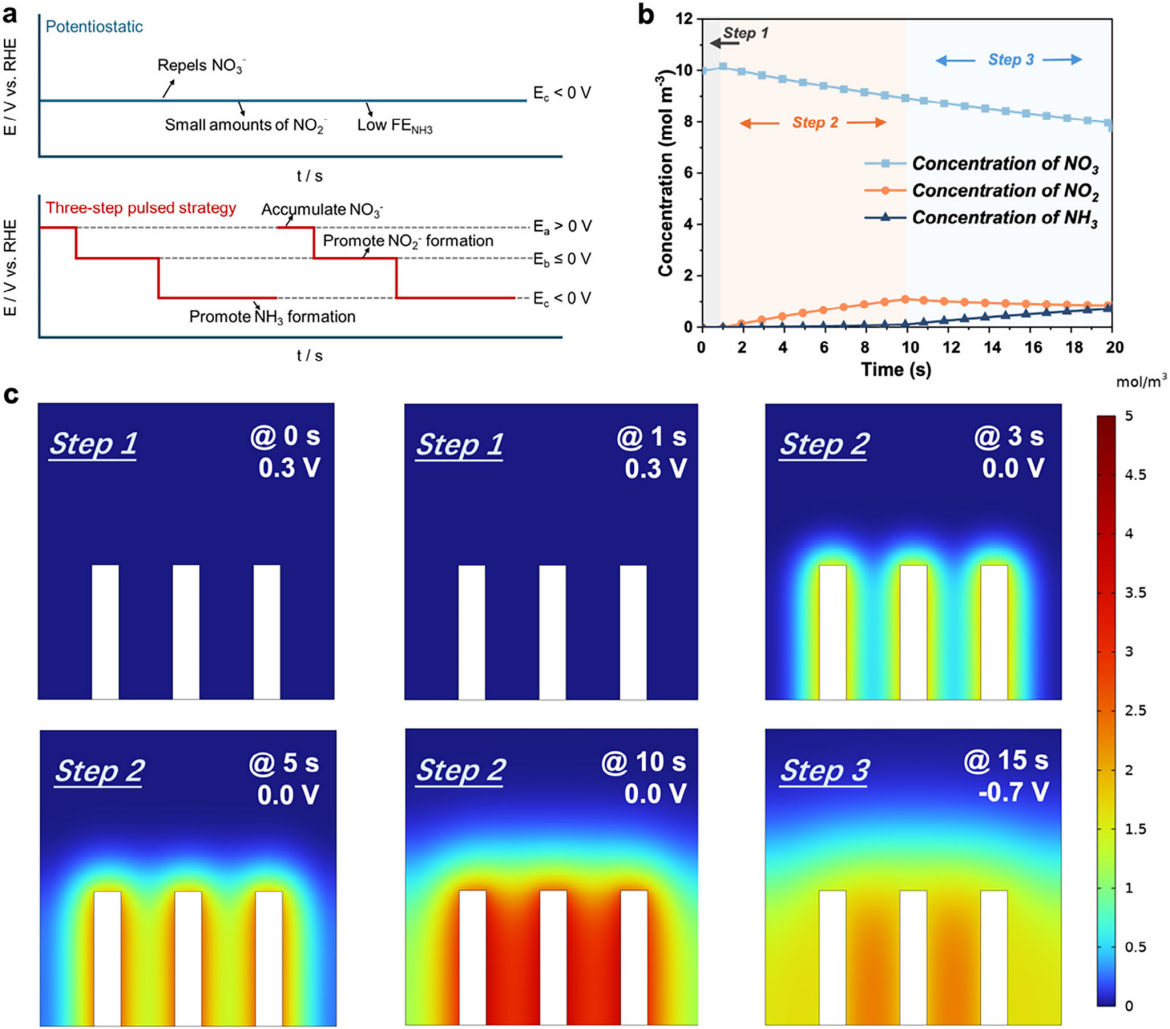
Simulations of 3‐step pulse NO_3_RR. a) Schematic of the ionic distribution along with the steps. b) Surface concentration simulations of NO_3_^−^, NO_2_^−^, and NH_3_ with time c) Snapshots of NO_2_^−^ concentrations at different reduction times.

Herein, we could basically conclude the mechanism as depicted in **Figure** **5**a. Under the condition of constant potentiostatic, the large negative bias potential would repel the NO_3_^−^ ions. Meanwhile, the poor S1 kinetics lead to the insufficient generation of key intermediate NO_2_^−^, which finally results in the low NH_3_ FE. In contrast, through the three‐step pulsed strategy, the NO_3_^−^ ions would first be accumulated at the electrode surface. The following two‐step potentials promote the NO_2_^−^ formation and the final conversion efficiency to NH_3_, respectively.

To further consolidate our proposed mechanism, the modeling of the tandem NO_3_^−^ accumulation/conversion process was performed in COMSOL Multiphysics software with a diluted species transport module. A geometric model including planar electrodes and electrolyte regions was established. In this model, the electrode is simplified into a continuous square area to reflect the reaction between the electrode surface and the solid–liquid interface of the electrolyte, while ignoring the changes inside the electrode. The domain equation is the diffusion equation, known as Fick's 2nd law, which describes the chemical transport of the electroactive species. At the bulk boundary, a uniform concentration is assumed to be equal to the bulk concentration for the reactants, where the products have zero concentration. At the electrode boundary, the reactant species are reduced to form the products. The specific parameters for numerical simulation have been provided in Table S5 (Supporting Information). For the external boundary, the top boundary is an electrolyte potential boundary, and the rest of the boundary conditions are insulation boundary conditions. The internal electrode boundary is set as the electrode surface, reflecting the process in which the reactant species are reduced to form the products. In the initial situation the bulk concentration is a uniform concentration is assumed to be equal to the bulk concentration for the reactants, where the products have zero concentration. The NO_3_RR process at the electrode–electrolyte interface could be separated into two reactions, which are NO_3_^−^ to NO_2_^−^ (2e^−^ process) and NO_2_^−^ to NH_3_ (6e^−^ process). Besides, the main side reaction HER was considered as well. Based on the modeling results, we obtained a surface concentration profile of NO_2_^−^ intermediates, NO_3_^−^ reactants, and NH_3_ products with time under pulsed conditions and constant electrolysis (Figure 5b). Here, we simulated according to our previously optimized step‐time profile as 1s‐10s‐20s for the three steps. It was shown that during Step 1 of the pulsed NO_3_RR, the concentration for NO_2_^−^ and NH_3_ remained zero, while a slight increasement in NO_3_^−^ concentration could be observed. After that, the NO_3_^−^ concentration continued to decline with time, which was the continuous nitrate consumption process in NO_3_RR. The concentration of NH_3_ first increases slightly at a relatively slow rate in Step 2. After 11 s, a clear inflexion point appeared at the start of Step 3, illustrating the enhanced reaction rate for the conversion to ammonia. To be mentioned, the intermediates NO_2_^−^ experienced an increasing and then decreasing trend, of which the peak concentration at the electrode surface was reached at the end of Step 2. The snapshots of NO_2_^−^ concentrations at different reduction times could intuitively suggest this trend as in Figure 5c. Besides, the simulated snapshots for the concentration of NO_3_^−^ and NH_3_ trend with time were provided in Supporting Information as well (Figures S17 and S18, Supporting Information).

To directly investigate HER kinetics under pulsed operation, we performed density functional theory (DFT) calculations (Figures S19 and S20, Supporting Information). Specifically, we calculated the energy barrier for HER on the Cu (111) surface, both in the presence and absence of *NO_2_ and *NO_3_ intermediates. The results show that the presence of *NO_2_ and *NO_3_ would significantly increase the energy barrier for H_2_ formation, from 0.06 to 0.09 and 0.21 eV, respectively. This mechanistic insight demonstrates that intermediate coverage during the pulse sequence intrinsically suppresses HER.

### 2.5 Prospects for Practical Applications and TEA Outlook

**Figure 6:**
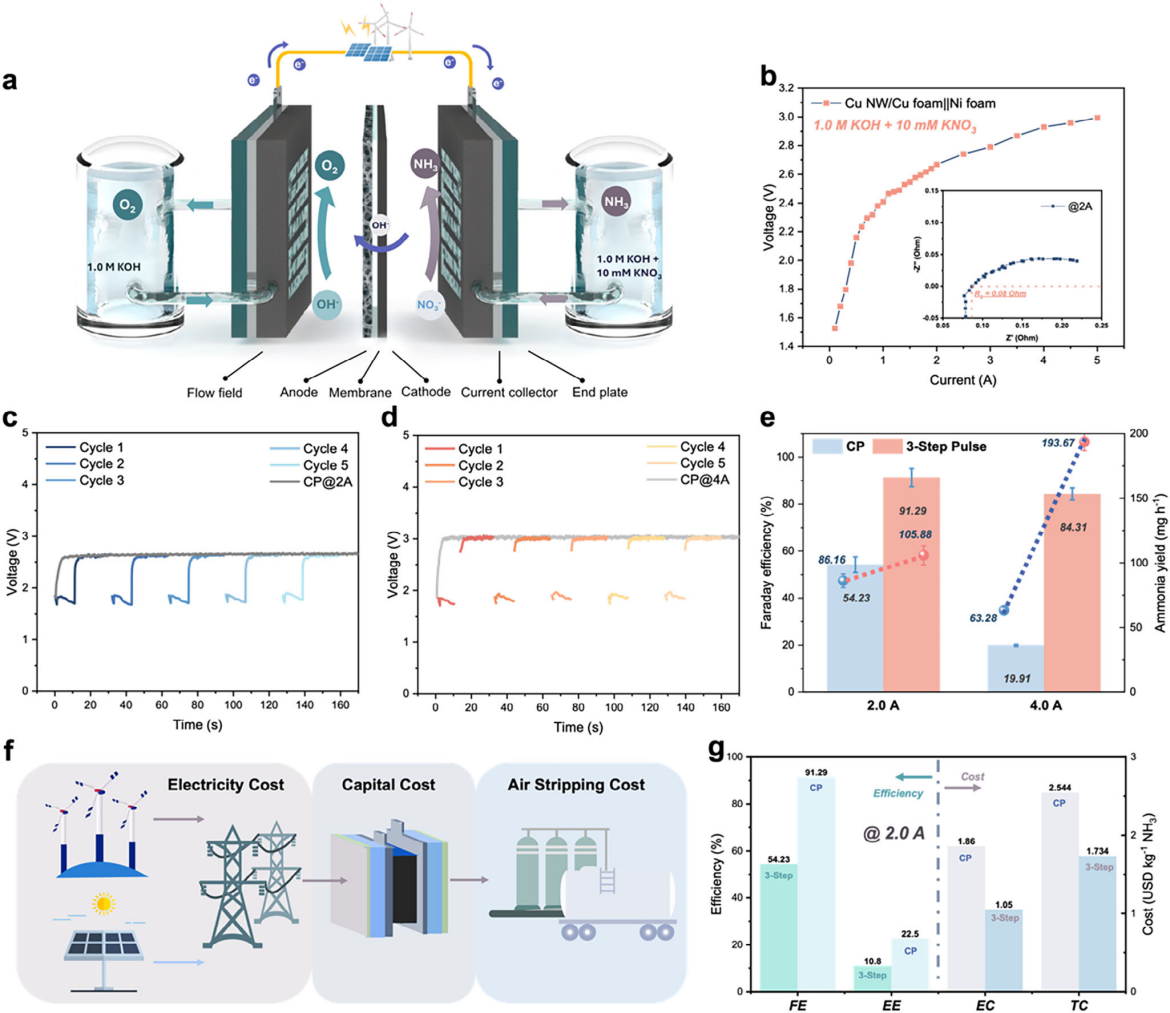
Application and TEA of 3‐step pulse NO_3_RR in a flow cell. a) Schematic of the flow cell. b) Voltage–current relationship (inset: Nyquist plot of the flow cell at 2.0 A). *V*–*t* curves of CP and 3‐step pulse NO_3_RR at c) 2.0 A and d) 4.0 A. e) Comparison of ammonia yield rate and corresponding Faraday efficiency between CP and 3‐step pulse NO_3_RR. f) Schematic of the cost breakdown for simplified TEA analysis. g) TEA comparison of CP and 3‐step pulse NO_3_RR applying the protocol reactor of 20 cm^2^ at 2.0 A.

The advantage of the three‐step pulsed NO_3_RR enables further exploration at the practical level facing the ultralow nitrate level and the high ammonia yield rate requirements. Here, we assembled a scaled‐up membrane electrode assembly (MEA) reactor for the continuous nitrate conversion to ammonia. The schematic of the MEA reactor is shown in **Figure** **6**a. Specifically, the MEA reactor's geometric active area was configured to 4.0 × 5.0 cm^2^. The Cu NW/Cu foam electrode served as the cathode, while a commercial Ni foam electrode functioned as the anode, with either an anion exchange membrane or a bipolar exchange membrane providing separation. The thickness of the cathode was determined by the Cu foam utilized (1.0 mm). On the cathode side, there was a continuous supply of a mixture of 1.0 m KOH and 10 mm KNO_3_ for nitrate reduction, using a syringe pump. Similarly, the anode side was circulated with 1.0 m KOH for water oxidation, also controlled by a syringe pump. The electrochemical performances of the reactor, such as electrolytic cell impedance and voltage–current curves, were investigated at first through the direct current power supply and electrochemical workstation (Figure 6b). To be mentioned, the three‐step pulsed voltages, based on the optimized parameters from the previous H‐cell experiment, were adjusted to the parameters of current in response to reaction conditions to enhance electrochemical performance. Specifically, the currents for the first step and the second step were controlled as −0.1 A (the minus sign means the opposite current direction) and 0.2 A. The currents for the third step of the pulse process were chosen as 2.0 and 4.0 A. The duration for the three steps was consistent with our previous Pulse‐2 protocol in the H‐cell experiment, which are 1s, 10s, and 20s, respectively. The corresponding *V*–*t* curves are shown in Figure 6c,d. Both the anode and cathode flow rates were maintained at 10 mL min^−1^. Besides, the NH_3_ molecules produced at the cathode were transported by the cathode flow and collected to assess the performance of nitrate reduction to ammonia. Here, we analyzed the ammonia FE and energy efficiency (EE) under two typical ammonia yield currents at 2.0 and 4.0 A. Compared to the CP electrolysis, the three‐step pulsed strategy enables the multiplication of the ammonia FE and the milligram‐level ammonia yield rate. At 2.0 A (100 mA cm^−2^), the highest FE for ammonia reached 91.29% and meanwhile, the ammonia yield rate was 105.88 mg h^−1^. At 4.0 A (200 mA cm^−2^), the highest FE for ammonia realized a fourfold increasement to 84.31% and the ammonia yield rate reached 193.67 mg h^−1^.

With the promising performance of the pulsed nitrate reduction in the MEA reactor, a simplified techno‐economic analysis (TEA) was conducted to evaluate its feasibility and whether this strategy is worth further commercialization and practical for real‐world application. In light of the enhanced performance of our scaled‐up MEA reactor and the data obtained from prior experimental investigations, a 10 mm nitrate concentration has been chosen as the influent electrolyte at the cathode. Meanwhile, the ammonia yield current was set to be 2.0 A for both the CP and the three‐step pulse. The subsequent calculations are predicated on the efficiency and yield metrics obtained during the experimental process. As shown in the schematic of cost breakdown in Figure 6f, we mainly considered the electricity cost, capital cost, and the air stripping cost in our TEA. First, the energy efficiency (EE) was calculated according to the Faraday efficiency, the ratio between cell voltage and theoretical voltage. Through applying the pulsed strategy, the EE increased from 10.8% to 22.5%. Second, we calculated the electricity cost (EC) for producing 1 kg of ammonia referencing the global weighted average levelized cost of energy for onshore wind electricity production. The EC decreased from 1.86 to 1.05 USD kg^−1^ applying the pulsed strategy. Furthermore, after evaluating both the capital and air‐stripping costs—the latter being the most commonly reported method for ammonia extraction, we calculated the total cost of producing ammonia via pulse NO_3_RR at 2.0 A.^[^ ^35^ ^]^ The estimated cost of this green ammonia is 1.73 USD kg^−1^. In comparison, the current market price for ammonia is 1.15 USD kg^−1^, indicating that nitrate reduction for ammonia production is not as economically competitive as the traditional Haber–Bosch process.^[^ ^36^ ^]^ However, when considering the potential savings from typical nitrate wastewater treatment costs (65 USD per kmol‐N for nitrate removal, equivalent to 3.82 USD kg^−1^ of NH_3_), our approach, which integrates the treatment process with ammonia coproduction, could be economically viable.^[^ ^37^ ^]^ In this context, our TEA underscored the potential of sustainable electrochemical processes as a feasible alternative to conventional local wastewater treatment facilities and the Haber–Bosch process. Furthermore, our three‐step approach enables the direct reduction of wastewater with low nitrate concentrations, thereby obviating the necessity for an additional nitrate concentration step. For future scale‐up to industrial level volumes, critical priorities include: a) reactor stacking for over 100‐fold electrode area amplification, b) integration with renewable energy sources, and c) validation of copper catalyst durability beyond 10 000 h of operation.

## 3 Conclusion

In summary, our work presented an efficient three‐step pulsed strategy to optimize the nitrate reduction to ammonia under ultralow concentrations of less than 10 mm. Through the bias potential modulation of the microenvironment of reactants (NO_3_^−^) and key intermediate (NO_2_^−^) at the electrode surface, the obstacle of the RDS step for NO_3_RR was solved and HER was greatly prohibited compared to the common CP strategy. The boosted improvement in ammonia production was observed with the highest NH_3_ FE reaching 99.84% at −0.7 V versus RHE. In addition, the underlying mechanisms of pulsed optimization were explored using a combination of operando characterizations and simulation validations. Implementing pulsed reduction in a flow cell achieved an ammonia yield rate of 193.67 mg h^−1^, demonstrating the practical potential for converting nitrates directly from wastewater without the need for preconcentration. Further, TEA confirmed the economic effectiveness of this pulsed method for ammonia production and highlighted the significant potential of sustainable electrochemical processes as a viable alternative to traditional local wastewater treatment facilities and the Haber–Bosch process. Overall, electrochemical nitrate reduction represents a promising technology for the efficient conversion of wastewater into valuable products.

## Conflict of Interest

The authors declare no conflict of interest.

## Author Contributions

L.A. X.Z. proposed the topic. K.Z. designed and conducted the experiment and drafted the manuscript. G.L. designed and conducted the computational simulation. All authors contributed to the editing of the manuscript.

## Supporting information



Supporting Information

## Data Availability

The data that support the findings of this study are available in the Supporting Information of this article.
