# Supplementary material for: Three‐Step Pulse Strategy Enhances Ultradilute Nitrate‐to‐Ammonia Conversion via Microenvironment and Mass Transfer Control
Source: Adv Sci (Weinh). 2025 Jul 28;12(40):e07720. doi: 10.1002/advs.202507720 (PMC12561188; doi:10.1002/advs.202507720)
Supplement: Supplementary file 1 — Supporting Information [file ADVS-12-e07720-s001.docx]

**Three-Step Pulse Strategy Enhances Ultra-Dilute Nitrate-to-Ammonia Conversion via Microenvironment and Mass Transfer Control**

Kouer Zhang^a^, Gang Liu^a^, Qing Wang^a^, Xiaoyu Huo^a^, Xiaohong Zou^a^, Mingcong Tang^a^, Xiao Zhang^a, b, *^ and Liang An^a, b, *^

^a^ Department of Mechanical Engineering, The Hong Kong Polytechnic University, Hung Hom, Kowloon, Hong Kong SAR, China

^b^ Research Institute for Smart Energy, The Hong Kong Polytechnic University, Hung Hom, Kowloon, Hong Kong SAR, China

*Corresponding authors:

Email: [xiao1.zhang@polyu.edu.hk](mailto:xiao1.zhang@polyu.edu.hk) (X. Zhang)

[liang.an@polyu.edu.hk](mailto:liang.an@polyu.edu.hk) (L. An)

**Experimental section**

**Reactants:**

Anhydrous ethanol (CH_3_CH_2_OH), and hydrochloric acid (HCl) were purchased from Anaqua (ACS reagent). Sodium hydroxide (NaOH), ammonium persulphate ((NH_4_)_2_S_2_O_8_), and potassium nitrate (KNO_3_) were purchased from Sigma-Aldrich (ACS reagent, ≥99.0%). All reactants were used without any further purification. All aqueous solutions were prepared with deionized water (resistivity over 18 MΩ cm at 25 °C, Millipore).

**Electrode fabrication:**

Firstly, the commercial Cu foam was first cut into the size of 1 cm $\text{×}$ 2 cm, and then washed with anhydrous ethanol and 1.0 M hydrochloric acid through ultrasonic method. Secondly, the Cu foam was soaked in 0.1 M ammonium persulfate and 1.0 M sodium hydroxide solution for 1 hour at room temperature. This method enabled in situ etching to grow uniform Cu(OH)_2_ nanowires (NWs) directly from the Cu foam. Then, the samples were annealed in a flowing 5% H_2_/Ar atmosphere with a flow rate of 100 sccm at 200 °C for 2 hours to synthesize Cu NW/Cu foam.

**Material characterizations:**

X-ray diffraction analysis (XRD) data were collected on a Rigaku Smart Lab X-ray diffractometer with Cu Kα radiation (Rigaku SmartLab 9kW). Scanning electron microscopy (SEM) was performed on TESCAN MIRA, which is a high-resolution (HR) analytical SEM with a high-brightness field emission electron source (FEG). Energy-dispersive X-ray spectroscopy (EDS) elemental mapping of SEM was performed on Tescan VEGA3 with energy dispersive X-ray spectroscopy (EDX) detector. Transmission electron microscopy (TEM), high-resolution TEM, HAADF-STEM, high-resolution HAADF-STEM and EDS elemental mapping were performed using a multipurpose JEOL JEM-2100F with field emission of 200 Kev. X-ray photoelectron spectroscopy (XPS) data were measured on Thermo Fisher Scientific Nexsa using monochromatic Al Kα radiation (1,486.6 eV). The XPS spectra were calibrated based on the detected carbon C 1s peak to 284.6 eV.

**Electrochemical characterizations:**

The electrochemical performance tests were first carried out in an H-cell using Fumasep FAA-3-50 as the ion-exchange membrane. In this three-electrode system, the samples synthesized in this work (1.0 cm $\text{×}$ 1.0 cm) were chosen as the working electrode (cathode), while the Pt foil (1.0 cm $\text{×}$ 1.0 cm) was chosen as the counter electrode (anode) and Hg/HgO electrode (with 1.0 M KOH solution) was chosen as the reference electrode. The catholyte was 1.0 M KOH and 10 mM KNO_3_ for the cathode, while the anolyte remained 1.0 M if not specified. The electrochemical performance data for the three-electrode system were collected and analyzed through the CHI 660e workstation. All the potentials measured against the Hg/HgO electrode in this work were converted to the reversible hydrogen electrode ($\text{RHE}$) scale through the equation below:

$$\text{E}\left( \text{ vs. RHE} \right)\text{ = E}\left( \text{ vs. Hg/HgO} \right)\text{ + 0.098 V + 0.0591 × pH}$$

The pH value of the 1.0 M KOH solution used in this work is 14.0 determined by pH meter (LICHEN pH-100A). The linear sweep voltammetry (LSV) was adopted with 90% IR-compensation of potential. The inner resistance was measured by the potentiostatic electrochemical impedance spectroscopy (EIS) at frequencies from 0.1 Hz to 100 kHz. The electrochemical active surface area ($\text{ECSA}$) was determined through the estimation of electrochemical double-layer capacitance (C_dl_) of the catalytic surface. The $\text{ECSA}$ was calculated according to the following equations:

$$\text{ECSA = }\frac{\text{C}_{\text{dl}}}{\text{C}_{\text{s}}}$$

$$\text{C}_{\text{dl}}\text{ = }\frac{\text{i}_{\text{c}}}{\text{v}}$$

In the above equations, C_s_ represents the specific capacitance (C_s_ = 0.040 mF cm^-2^); it is the value of the charging current density (mA cm^-2^) which is half of the charge/discharge current difference; v is the scan rate (mV s^-1^). The cyclic voltammetry (CV) curves in C_dl_ determination were measured in a potential window without Faradaic current occurring in the electrolyte of 1.0 M KOH under incremental scanning rates of 20 mV s^-1^, 40 mV s^-1^, 60 mV s^-1^, 80 mV s^-1^, and 100 mV s^-1^, respectively.

**Product detection:**

The concentration of ammonia is decided through the technique of ultraviolet (UV) absorption spectroscopy. The indophenol blue spectrophotometric method was chosen in this work for ammonia detection. Three colour reagents were prepared in advance, which were named solution A, solution B and solution C. Solution A is a mixture of 1.0 M NaOH, 5 wt% salicylic acid and 5 wt% sodium citrate solution. Solution B is 0.05 M Sodium hypochlorite (NaClO) and solution C is 0.05 M sodium nitroferricyanide (C_5_FeN_6_Na_2_O). Before the test in UV, the sample electrolyte was diluted to the detection range. After that, 1.0 ml of dilution electrolyte was taken and 1.0 ml solution A, 0.5 ml solution B, and 0.1 ml solution C were added sequentially and placed in a light-proof environment for 2 hours. Finally, the solution was measured in UV-vis spectrophotometry (Dynamica Halo-DB20). To be mentioned, the standard concentration-absorbance curve was calibrated using different concentrations of standard NH_4_Cl solution (Figure S9). The peak for the formation of indophenol blue appeared at the wavelength of 655 nm.

In addition, isotope-labelling measurement was also adopted to prove the source of ammonia produced in this work. The ^1^H NMR (500MHz SB Liquid Bruker Advance NMR Spectrometer) method was chosen using D_2_O as solvent.

The concentration of nitrate was detected in this work through the technique of ultraviolet (UV) absorption spectroscopy. Firstly, samples were collected and diluted to the detection range. Next, 40.0 µl of 1.0 M hydrochloric acid and 4.0 µl of 0.8wt% sulfamic acid solution were added to 2.0ml of diluted sample. After placing for 30 minutes, the absorption intensities at wavelengths of 220 and 275 nm were recorded. The absorbance was calculated according to the formula below:

$$\text{A = A}_{\text{220nm}}\text{ – }\text{A}_{\text{275nm}}$$

The concentration-absorbance curve was calibrated using a series of standard potassium nitrate solutions which were linearly fitted. The concentration of nitrate was then calculated from the nitrate concentration-absorbance curve.

The concentration of nitrite was detected in this work through the technique of ultraviolet (UV) absorption spectroscopy. Similarly, samples were collected and diluted to the detection range. The colour agent for the detection of nitrite is a mixture of p-aminobenzene sulfonamide (8.0 g), N-(1-naphthyl) ethylenediamine dihydrochloride (0.4 g) in 100 ml of deionized water. Then, 40.0 µl of the prepared colour agent was added to 2.0 ml of the diluted electrolyte sample and rested for 30 min before being tested in UV and the adsorption peak appeared at 540 nm. The concentration-absorbance curve was calibrated using a series of standard potassium nitrite solutions which were linearly fitted. The concentration of nitrite was then calculated from the concentration-absorbance curve (Figure S10).

**Flow cell reactor fabrication and setup:**

The scaling-up flow cell (5 cm × 4 cm) was designed and fabricated as an industrialized prototype. In this design, endplates, current collectors, and flow fields were placed in sequence at outer sides of anode and cathode. The effective apparent area for both anode and cathode were 20 cm^2^. Similarly, serpentine flow channel was employed in flow field and gaskets fitting the size of the flow cell are used to prevent leakage. Fumasep FAA-3-50 was chosen as the AEM. The anode was the commercial Ni foam and the as-prepared Cu NW/Cu foam was employed as the cathode. Electrolyte was sent to the flow-cell reactor through peristaltic pumps at the flow rate of 10.0 mL min^-1^.The current was provided and recorded by the IT6500 DC power supply and its software (ITECH).

**Ammonia Faraday efficiency (FE), energy efficiency (EE) and yield rate calculations:**

The FE for ammonia production was calculated based on the following equations:

$$\text{FE =}\frac{\text{n × c × V × F}}{\text{Q}}$$

The yield rate of ammonia is calculated with the equation below:

$$\text{Y}_{\text{N}\text{H}_{\text{3}}}\text{ =} \frac{\text{c}}{\text{t}}$$

The corresponding EE of the flow-cell reactor is calculated as below:

EE = FE $\text{× }\frac{\text{U}_{\text{0}}}{\text{U}}$

In the above equations, n is the electron transfer number of the reaction (n = 8 for ammonia, n = 2 for nitrite in NO_3_RR; n = 6 for NO_2_RR to ammonia); cis the concentration of the product (mol mL^-1^); t is the reaction time (h); F is the Faraday constant (F = 96485 C mol^-1^); Vis the volume of cathode electrolyte in H-cell reactor and cathode electrolyte tank for flow-cell reactor (mL); Q is the total consumed amount of charge (C).

All experiments for key measurements were repeated three times if not otherwise specified.

**Methods for density functional theory calculations:**

All density functional theory calculations were performed by Vienna ab initio simulation package (VASP).^[1]^ The Perdew-Burke-Ernzerhof (PBE)^[2]^ functional was employed to treat the exchange-correlation interactions. The plane-wave basis set with a kinetic energy cutoff of 400 eV, the energy convergence criterion of 10^-5^ eV, the force convergence criterion of 0.02 eV Å^−1^, and a (2×2×1) Monkhorst-Pack k-point sampling was employed for structure relaxation. H_2_ and H_2_O were calculated in boxes of 20 Å×20 Å×20 Å with the gamma point only. The free energy diagrams for HER were calculated with reference to the computational hydrogen electrode.^[3]^ The free energy of gas phase and adsorbed species can be obtained from the following equation:

∆G=∆E_DFT_ + ∆ZPE -T∆S

where T was set as 298.15 K. ∆ZPE and TΔS was the change in the zero point energy and entropy.

**References:**

[1] G. Kresse, J. Furthmüller, Efficient iterative schemes for ab initio total-energycalculations using a plane-wave basis set, Phys. Rev. B 54 (1996) 11169.

[2] J.P. Perdew, K. Burke, M. Ernzerhof, Generalized gradient approximation madesimple, Phys. Rev. Lett. 77 (1996) 3865.

[3] J.K. Nørskov, J. Rossmeisl, A. Logadottir, L. Lindqvist, J.R. Kitchin, T. Bligaard, H. Jonsson, Origin of the overpotential for oxygen reduction at a fuel-cell cathode, J. Phys. Chem. B 108 (2004) 17886–17892.

**Finite element numerical simulation**

COMSOL Multiphysics 6.2 was used to numerically simulate the change of ion concentration during the nitrate reduction process under the change of pulse voltage. A two-dimensional grid model including planar electrodes and electrolytes is established. For potential-controlled two-step electrochemical reactions, the Nernst-Planck equation can well describe the distribution of current and potential due to diffusion and migration.

Where D represents the diffusion coefficient of the ion, c represents the concentration of the ion, and t represents time.

In the initial condition, the concentration of the entire electrolyte region is regarded as the concentration of the bulk electrolyte, and the concentration of the products is zero. The electrode reaction is assumed to occur at the electrode boundary. The opposite boundary of the electrode surface is assumed to be a constant electrolyte potential. Reactions that occur on the electrode surface include:

NO_3_^-^ + H_2_O + 2e^-^ → NO_2_^-^ + 2OH^-^

NO_2_^-^ + 5H_2_O + 6e^-^ → NH_3_ + 7OH^-^

The local current density at the electrode is mainly represented by the Butler-Volmer equation. The specific equations are as follows:

$$j_{NO_{3}^{-}}=j_{0,NO_{3}^{-}}\left[\left(\frac{c_{OH^{-}}}{c_{OH^{-},ref}}\right)^{2}\frac{c_{NO_{2}^{-}}}{c_{NO_{2}^{-},ref}}\exp\left(\frac{\alpha_{a}F\eta}{RT}\right)-\frac{c_{NO_{3}^{-}}}{c_{NO_{3}^{-},ref}}\exp\left(\frac{\alpha_{c}F\eta}{RT}\right)\right]$$

$$j_{NO_{2}^{-}}=nFk_{0,NO_{2}^{-}}c_{0,NO_{2}^{-}}\left[\left(\frac{c_{OH^{-}}}{c_{OH^{-},ref}}\right)^{7}\frac{c_{NH_{3}}}{c_{NH_{3},ref}}\exp\left(\frac{\alpha_{a}F\eta}{RT}\right)-\frac{c_{NO_{2}^{-}}}{c_{NO_{2}^{-},ref}}\exp\left(\frac{\alpha_{c}F\eta}{RT}\right)\right]$$

Where j is the local current density, $\alpha$is the transfer coefficient, F is the Faraday constant, R is the ideal gas constant, T is the reaction temperature, and $\eta$ is the reaction overpotential. Among them, the equation of reference local current density is as follows:

$$j_{0,NO_{3}^{-}}=nFk_{0,NO_{3}^{-}}c_{0,NO_{3}^{-}}$$

For NO_3_^-^, considering the effect of adsorption rate in the reaction, Langmuir isotherm was used to measure the adsorption kinetics.

$$r=k_{a}c_{0,NO_{3}^{-}}\left(1-\Theta_{NO_{3}^{-}}-\Theta_{NO_{2}^{-}}\right)-k_{d}\Theta_{NO_{3}^{-}}$$

Where $\theta$is the electrode surface coverage, k_a_ is the adsorption rate constant, and k_b_ is the desorption rate constant.

**Supplementary figures**


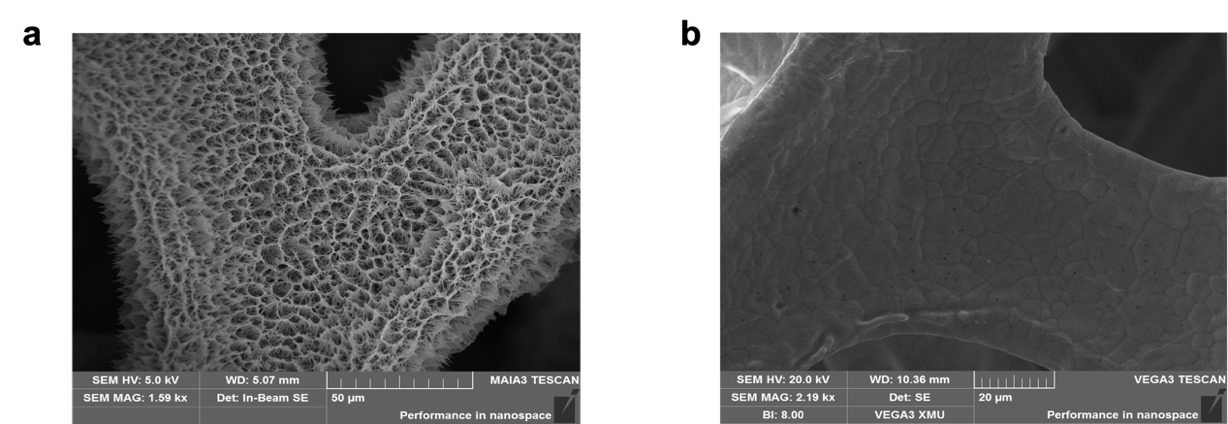


**Figure S1.** The SEM image of (a) Cu foam and (b) Cu NW/Cu foam.


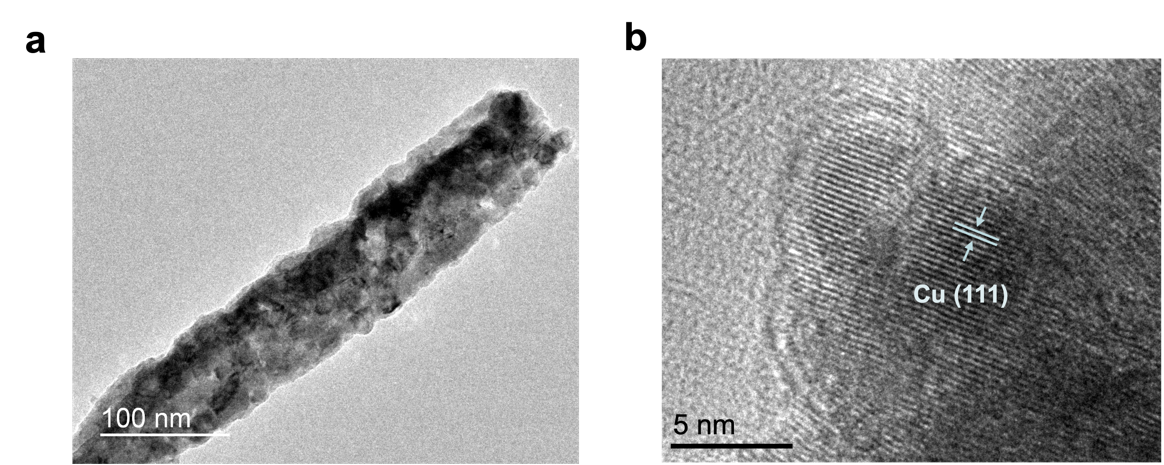


**Figure S2.** The (a) TEM and (b) HRTEM image of Cu NW/Cu foam.


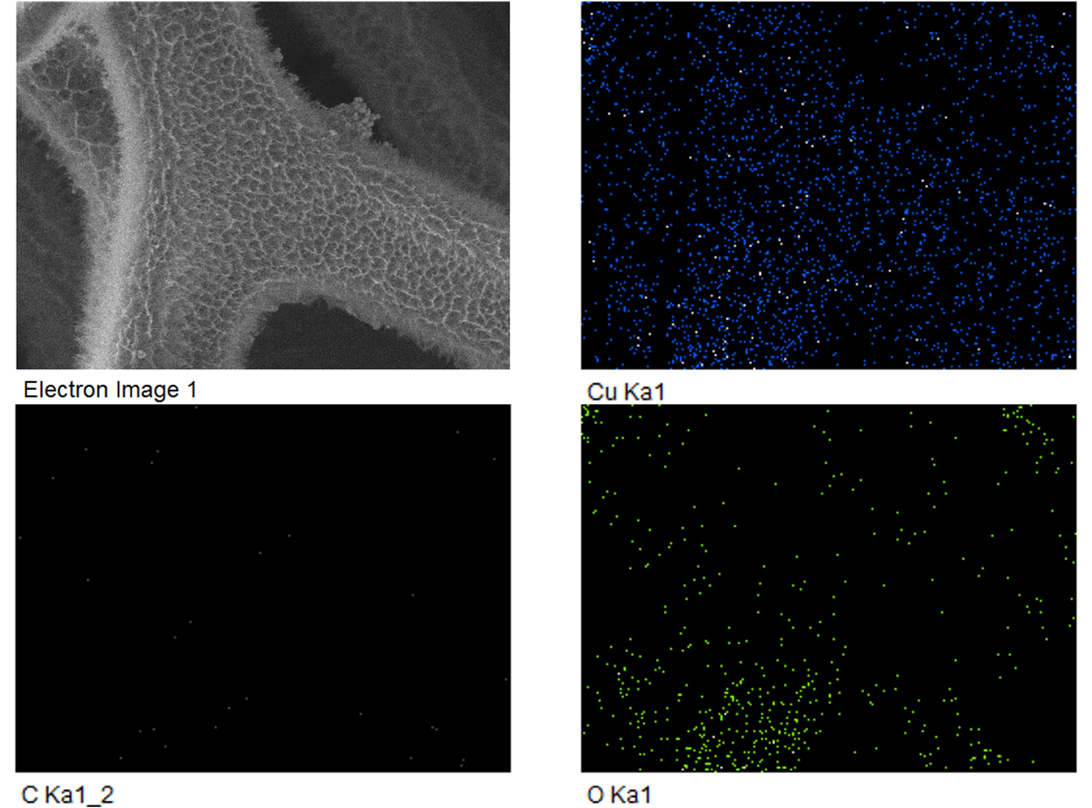


**Figure S3.** The SEM mapping of Cu NW/Cu foam.


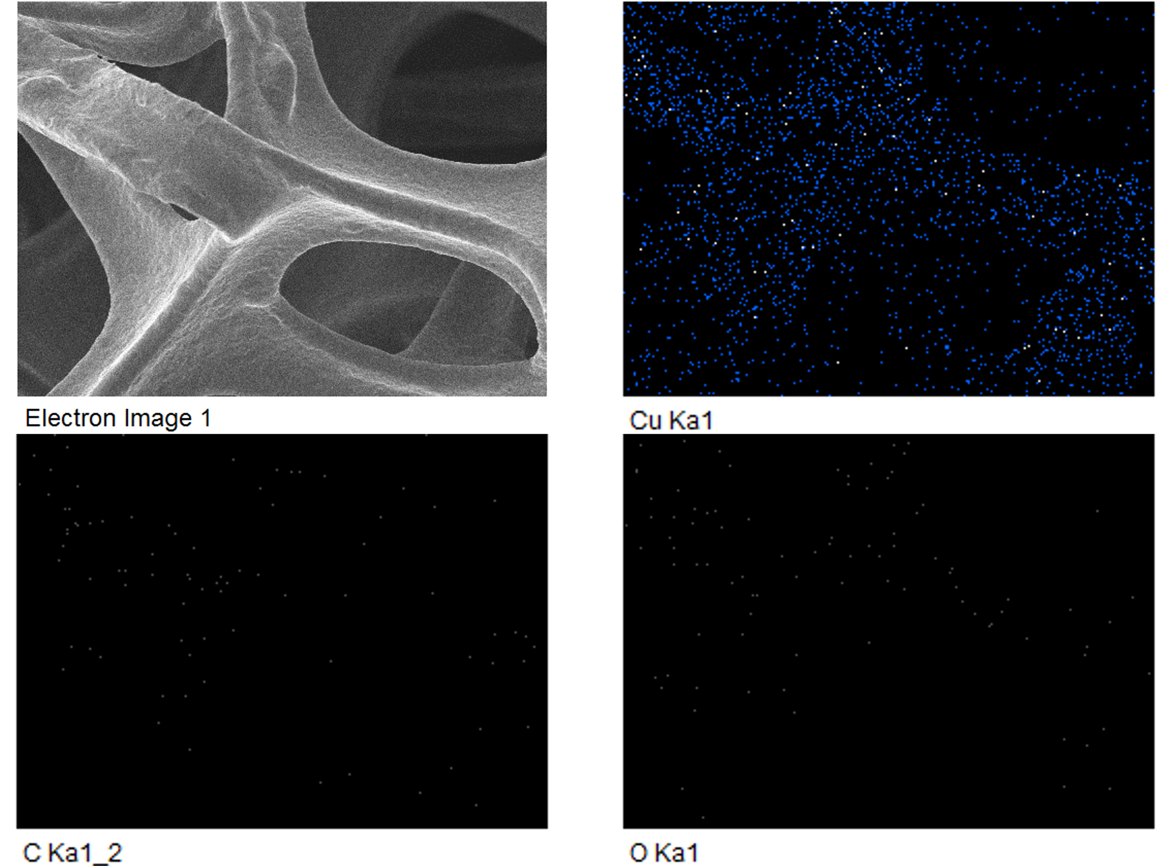


**Figure S4.** The SEM mapping of commercial Cu foam.


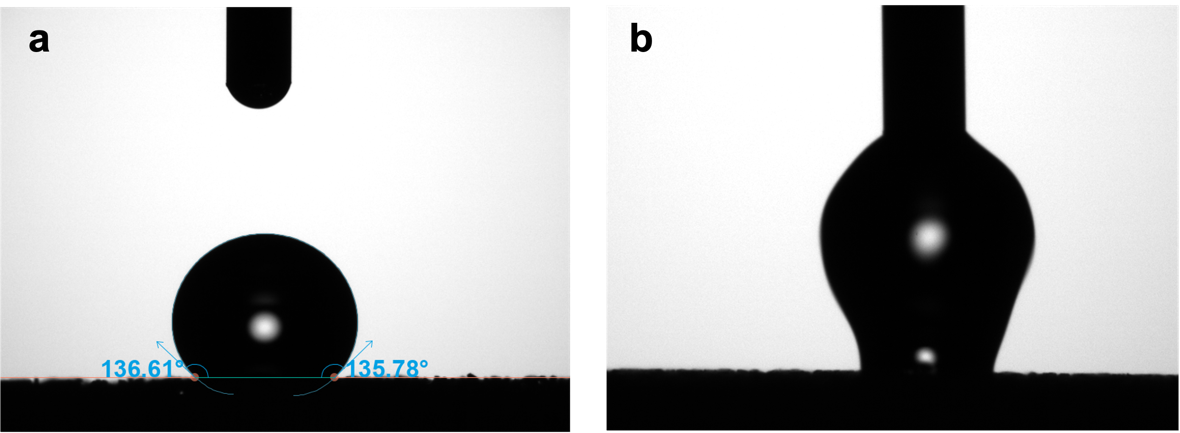


**Figure S5.** The contact angle of (a) Cu foam and (b) Cu NW/Cu foam


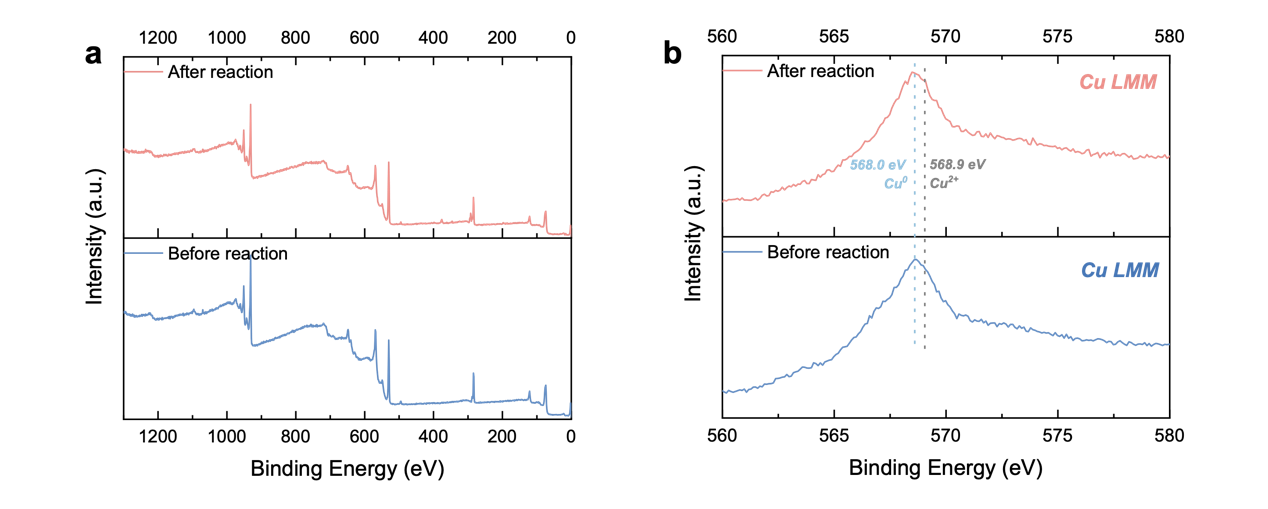


**Figure S6.** The XPS spectra of Cu NW/Cu foam before and after reaction including (a) survey scan and (b) Cu LMM.


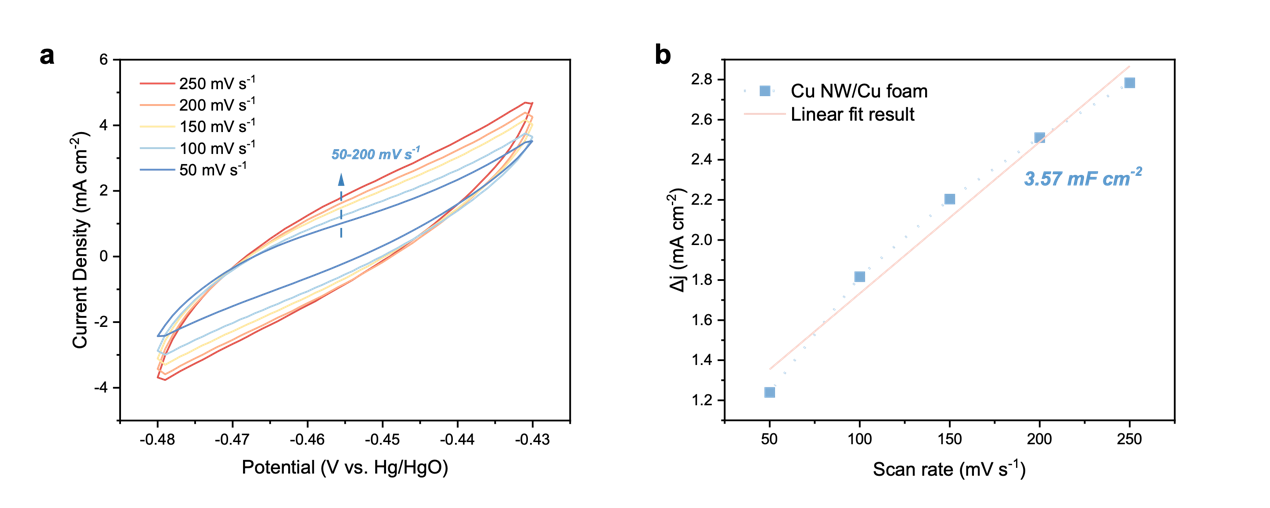


**Figure S7.** (a) CV scan under different scan rates and (b) ECSA of Cu NW/Cu foam.


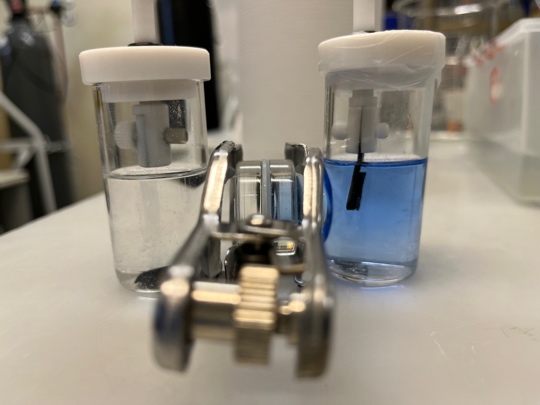


**Figure S8.** The photo of electrolyte after 5 hours reaction at 0.3 V vs. RHE.

**Figure S9.** LSV curves comparison of Cu NW/Cu foam and Cu foam in the electrolyte of 1.0 M KOH with 10 mM KNO_3_.


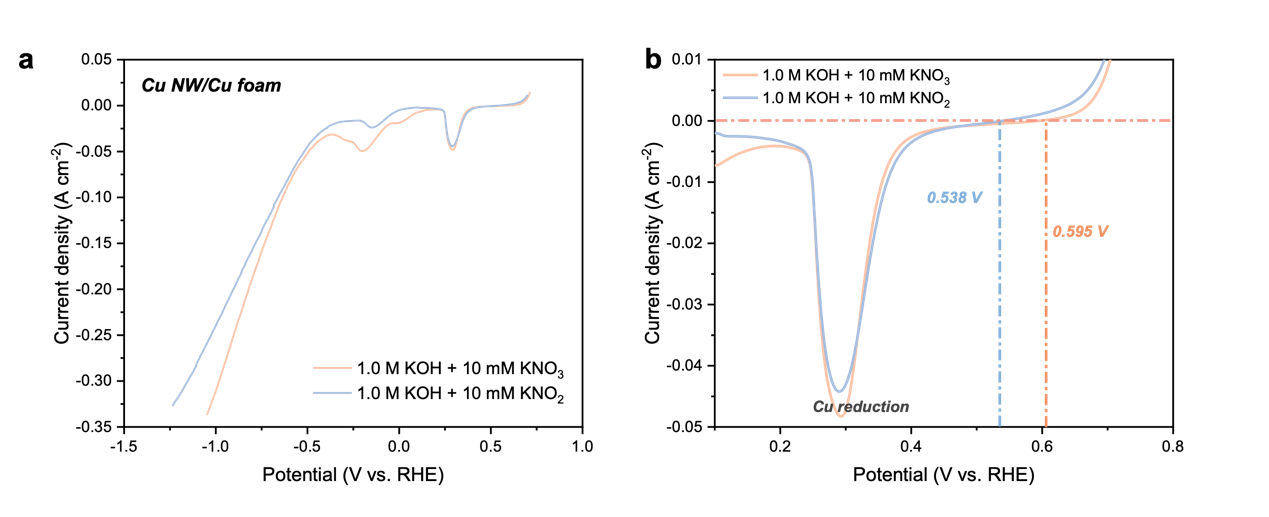


**Figure S10.** (a) LSV curves and (b) zoom-in curves of Cu NW/Cu foam in the electrolyte of 1.0 M KOH with 10 mM KNO_3_ and 10 mM KNO_2_.


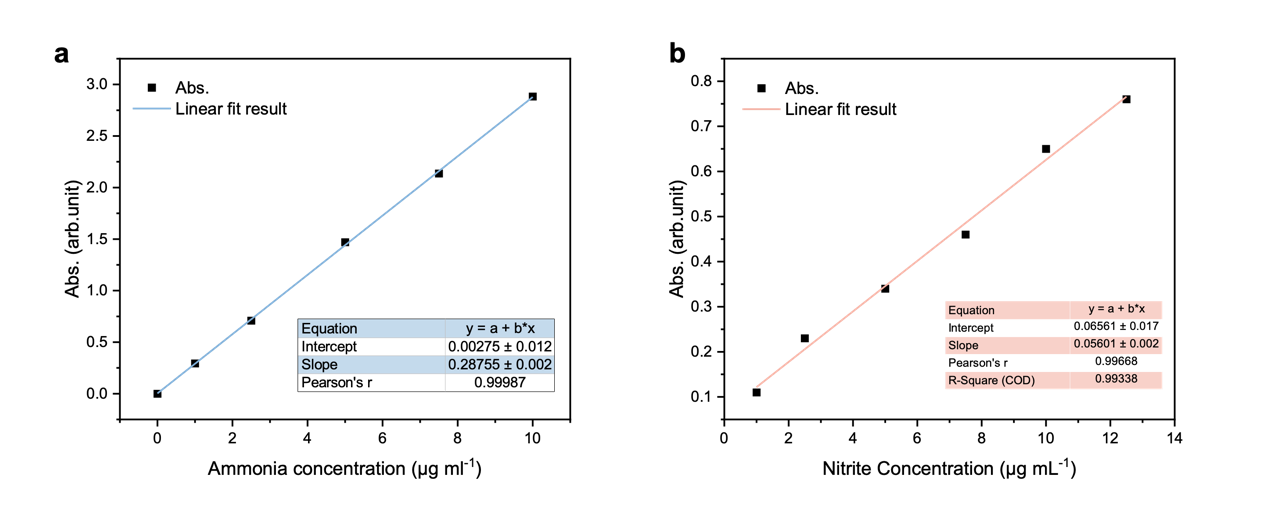


**Figure S11.** Concentration-absorbance linear standard curve of (a) ammonia and (b) nitrite.


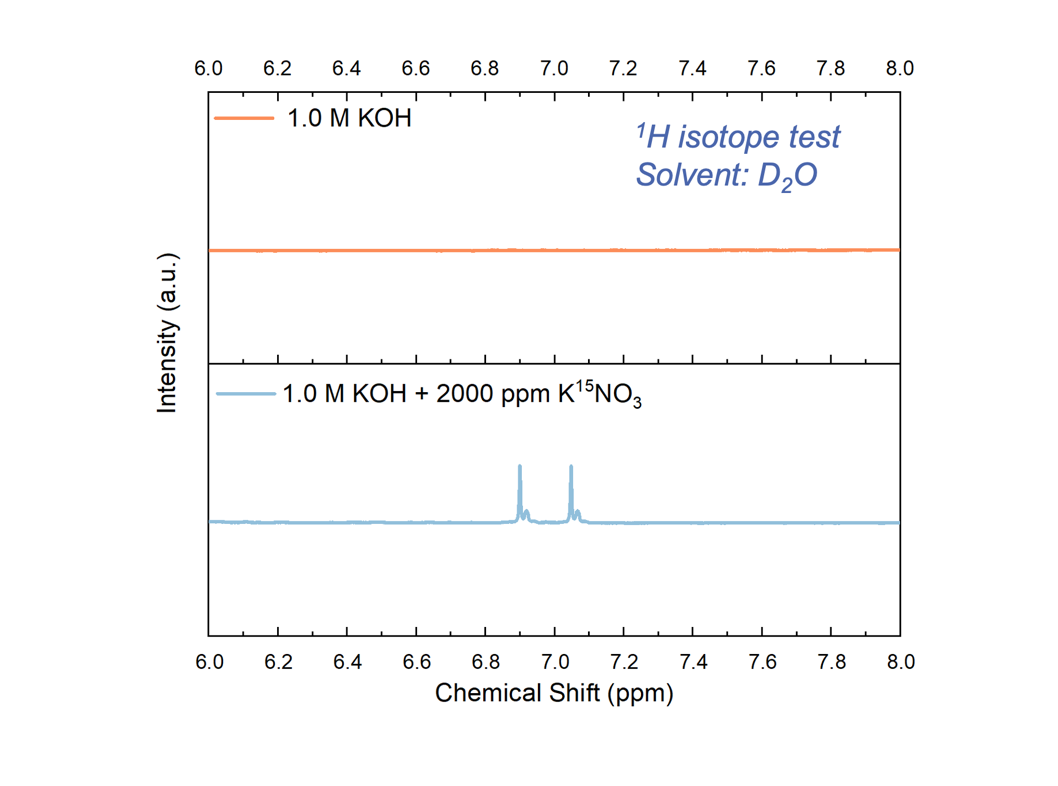


**Figure S12.** ^1^H NMR spectra of electrolyte after reaction using D_2_O as solvent.


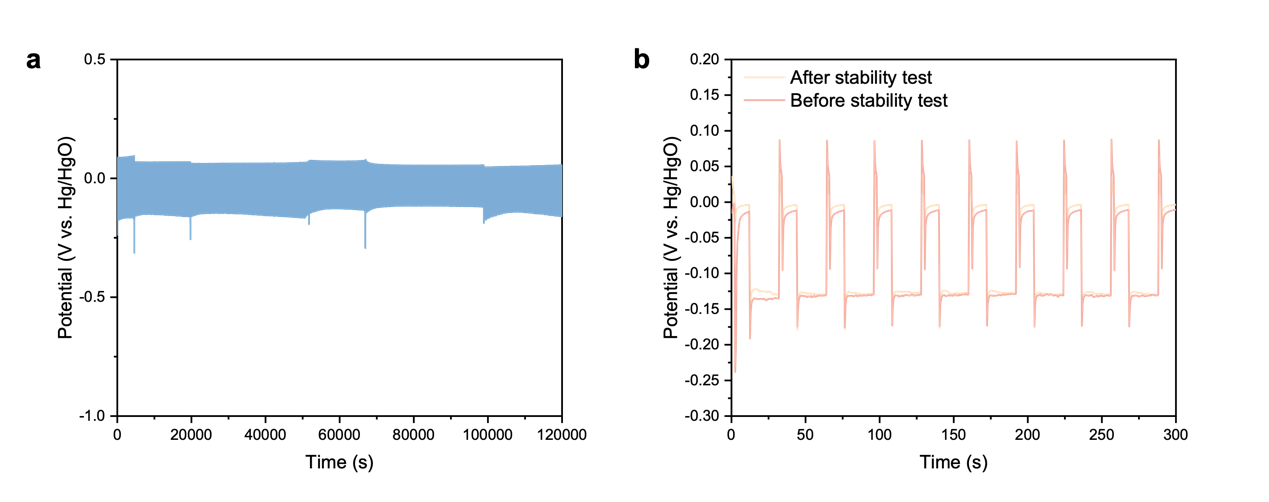


**Figure S13.** (a) V-t curve of stability test of 3-step pulsed NO_3_RR and (b) corresponding comparison of samples before and after stability test.


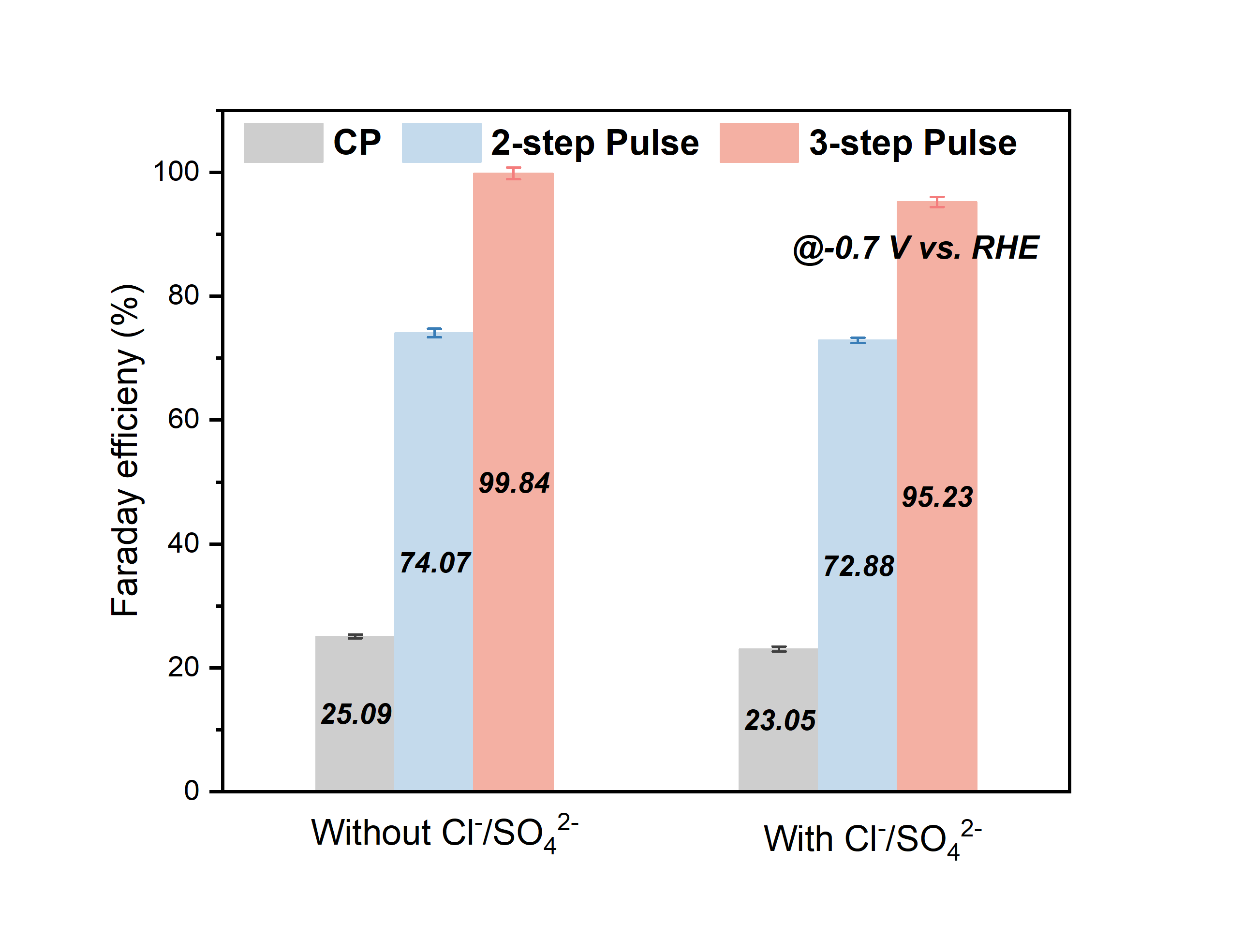


**Figure S14.** Ammonia Faraday efficiency of CP, 2-step pulse and 3-step pulse NO_3_RR -0.7 V vs. RHE with/without pollutant anions (Cl^-^ and SO_4_^2-^).

**Figure S15.** DEMS intermediates signals of N_2_O (m/z=44) under different reduction potentials and conditions.


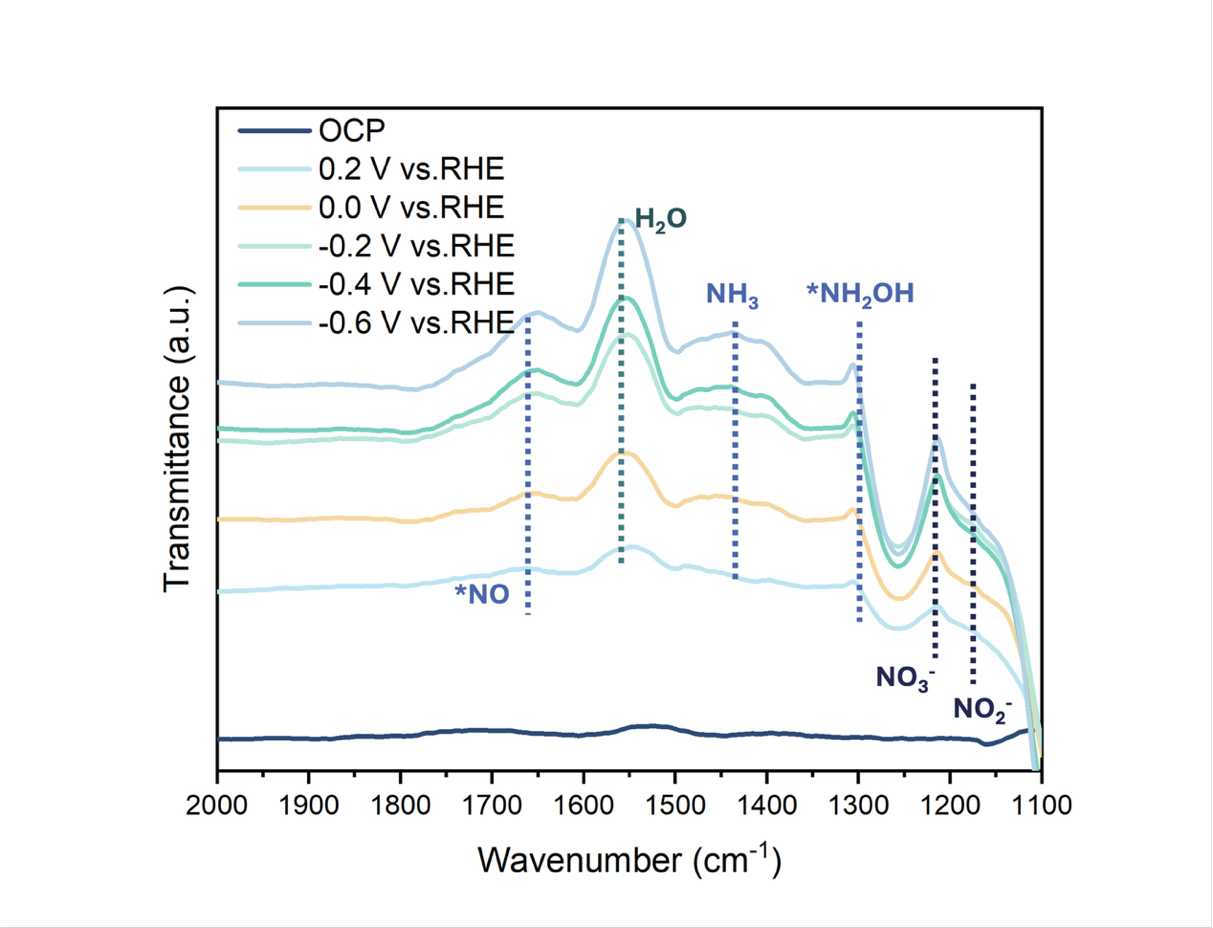


**Figure S16.** The In-situ FTIR spectra of Cu NW/Cu foam under various potential bias (OCP to -0.6 V vs. RHE)


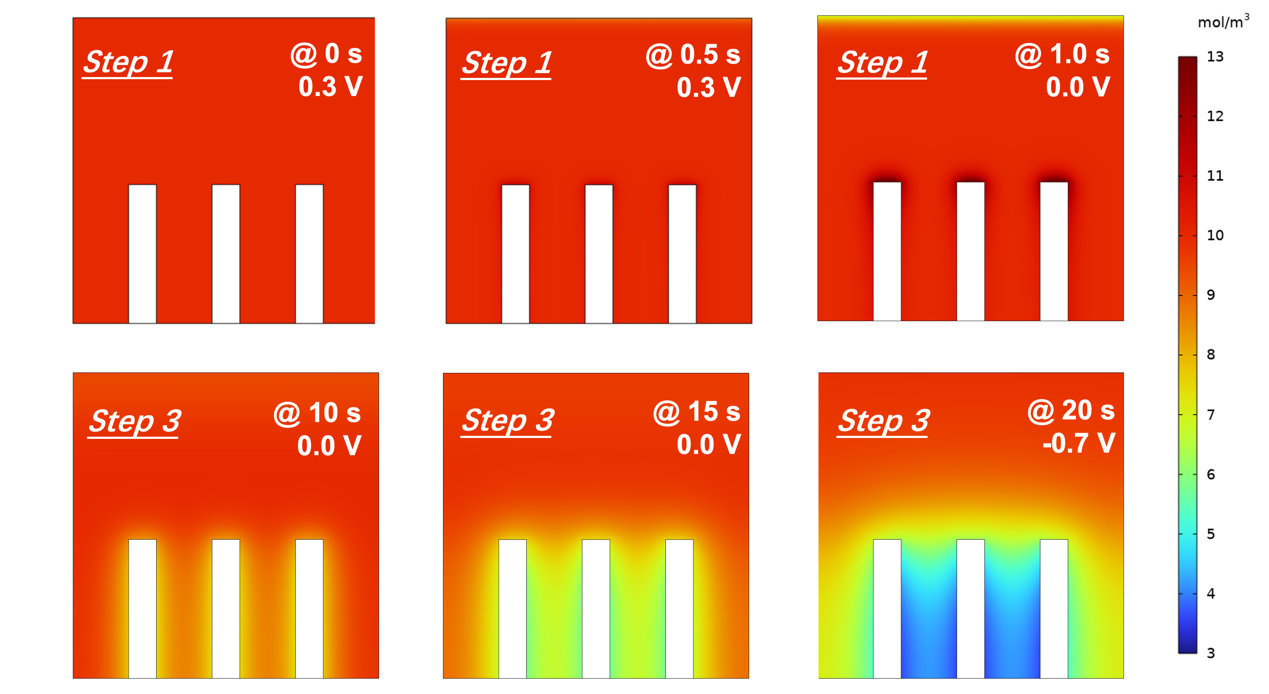


**Figure S17.** COMSOL simulation snapshots of NO_3_^-^ concentrations at different reduction times.


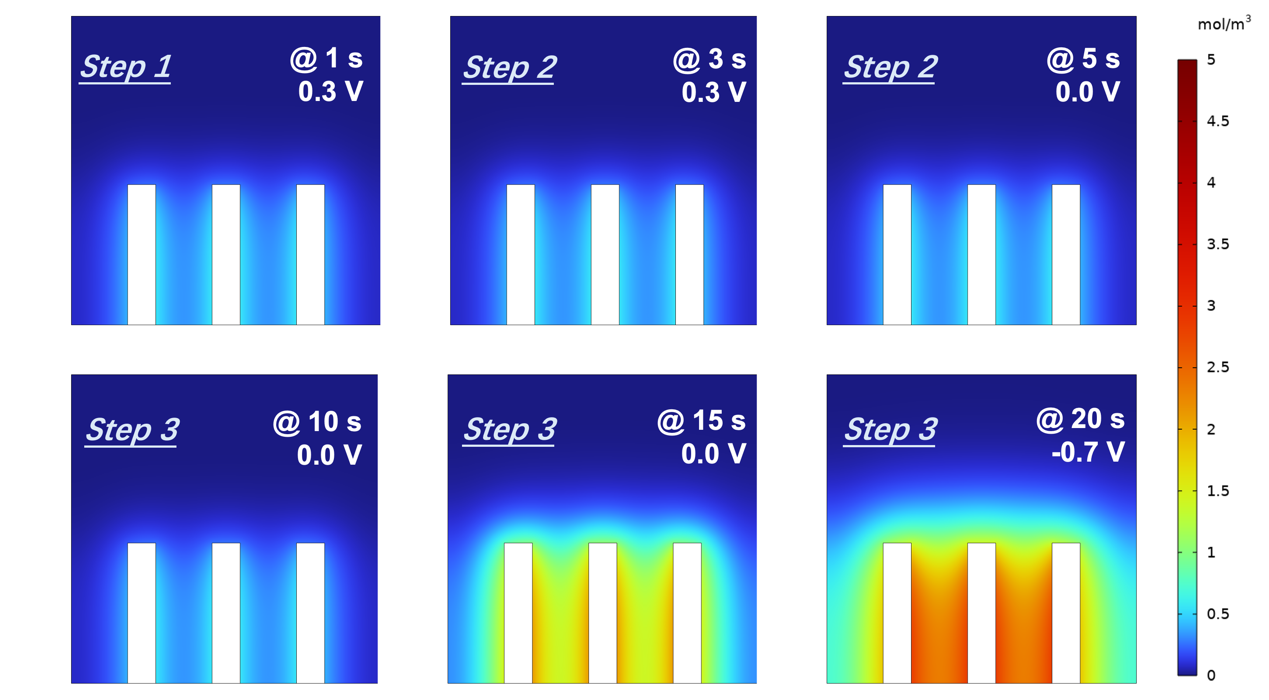


**Figure S18.** COMSOL simulation snapshots of NH_3_ concentrations at different reduction times.


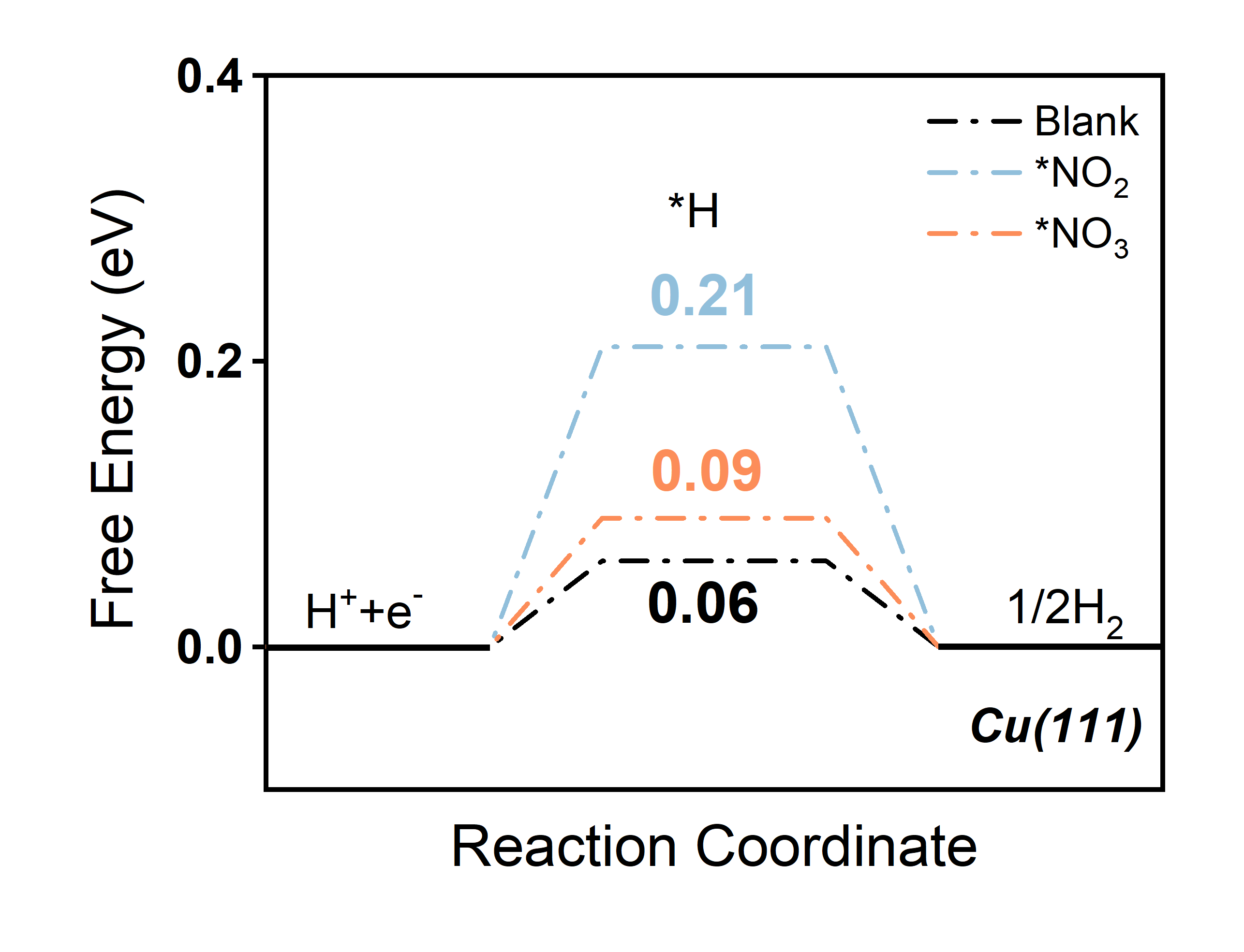


**Figure S19.** The calculated free energy diagram of the HER at the equilibrium potential for different absorbed ions on Cu(111) surface.


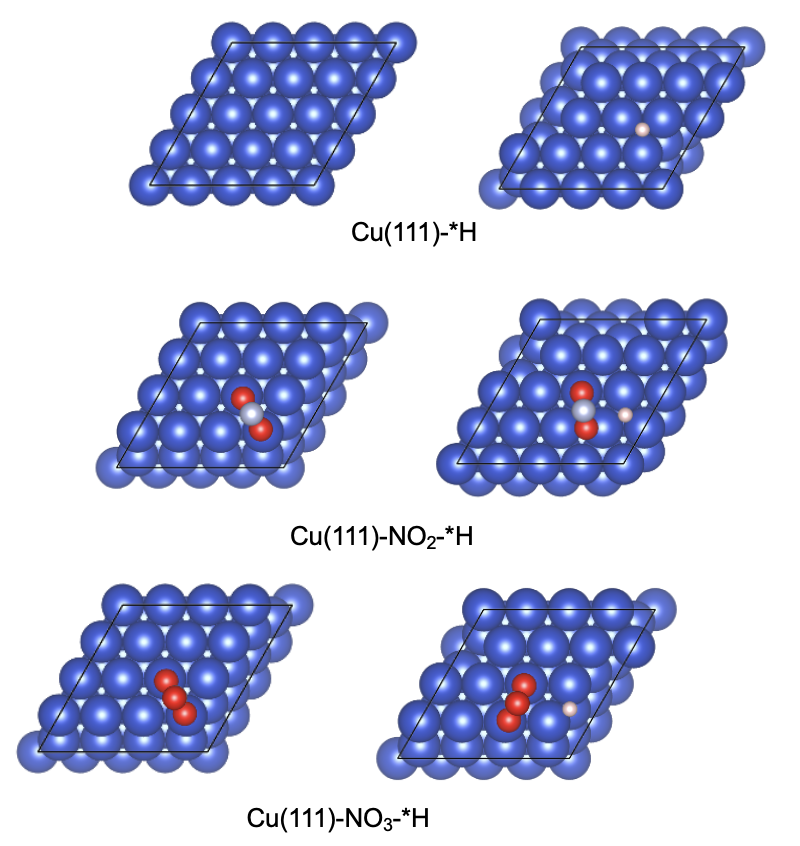


**Figure S20.** The absorbed models of the *H, *NO_2_ and *NO_3_ on Cu(111) surface for DFT calculations.

**Supplementary tables**

**Table S1.** XPS composition analysis of Cu NW/Cu foam before reaction

| **Before reaction** | **Position** | **Area** | **At%** |
| --- | --- | --- | --- |
| Cu^0^ 2p 3/2 | 932.2 | 85169.4 | 40.5% |
| Cu^2+^ 2p 3/2 | 934.4 | 38267.1 | 18.2% |
| Cu^0^ 2p 1/2 | 952.2 | 43745.4 | 20.8% |
| Cu^2+^ 2p 1/2 | 954.5 | 13212.3 | 6.28% |

**Table S2.** XPS composition analysis of Cu NW/Cu foam after reaction

| **After reaction** | **Position** | **Area** | **At%** |
| --- | --- | --- | --- |
| Cu^0^ 2p 3/2 | 932.2 | 60148.7 | 43.0% |
| Cu^2+^ 2p 3/2 | 934.4 | 25767.4 | 18.4% |
| Cu^0^ 2p 1/2 | 952.2 | 27291.0 | 19.5% |
| Cu^2+^ 2p 1/2 | 954.5 | 9948.4 | 7.11% |

**Table S3.** SEM mapping composition analysis of commercial Cu foam

| **Element** | **Weight%** | **Atomic%** |
| --- | --- | --- |
| **C K** | 12.52 | 43.10 |
| **Cu K** | 87.48 | 56.90 |
| **Totals** | 100.00 | 100.00 |

**Table S4.** SEM mapping composition analysis of commercial Cu foam

| **Element** | **Weight%** | **Atomic%** |
| --- | --- | --- |
| **O K** | 14.79 | 40.81 |
| **Cu K** | 85.21 | 59.19 |
| **Totals** | 100.00 | 100.00 |

**Table S5.** Main parameters for numerical simulation^[1,2]^

| **Parameters** | **Values** | **Units** | **Remarks** |
| --- | --- | --- | --- |
| D_NO3_^-^ | 1.9×10^-9^ | m^2^ s^-1^ | Diffusion coefficient |
| D_NO2_^-^ | 1.9×10^-9^ | m^2^ s^-1^ |  |
| D _OH_^-^ | 5.273$\times$10^-9^ | m^2^ s^-1^ |  |
| c_0, NO3_^-^ | 10 | mol m^-3^ | Initial ion concentration |
| c_0, NO2_^-^ | 0 | mol m^-3^ |  |
| c_0, OH_^-^ | 100 | mol m^-3^ |  |
| k_0, NO3_^-^ | 1.0×10^-4^ | m s^-1^ | reaction rate constant |
| k_0, NO2_^-^ | 1.0×10^-4^ | m s^-1^ |  |
| R | 8.314 | J mol^-1^ K^-1^ | ideal gas constant |
| T | 96485 | C mol^-1^ | Faraday constant |

**References:**

[1] Li, P., Li, R., Liu, Y., Xie, M., Jin, Z., & Yu, G. (2023). Pulsed nitrate-to-ammonia electroreduction facilitated by tandem catalysis of nitrite intermediates. *Journal of the American Chemical Society*, *145*(11), 6471-6479.

[2] Guo, J., Brimley, P., Liu, M. J., Corson, E. R., Muñoz, C., Smith, W. A., & Tarpeh, W. A. (2023). Mass transport modifies the interfacial electrolyte to influence electrochemical nitrate reduction. *ACS Sustainable Chemistry & Engineering*, *11*(20), 7882-7893.
